# Human Capital, Values, and Attitudes of Persons Seeking Refuge in Austria in 2015

**DOI:** 10.1371/journal.pone.0163481

**Published:** 2016-09-23

**Authors:** Isabella Buber-Ennser, Judith Kohlenberger, Bernhard Rengs, Zakarya Al Zalak, Anne Goujon, Erich Striessnig, Michaela Potančoková, Richard Gisser, Maria Rita Testa, Wolfgang Lutz

**Affiliations:** 1 Vienna Institute of Demography, Austrian Academy of Sciences, Vienna, Austria; 2 Department for Socioeconomics, Vienna University of Economics and Business, Vienna, Austria; 3 World Population Program, International Institute for Applied Systems Analysis, Laxenburg, Austria; Queensland University of Technology, AUSTRALIA

## Abstract

Since its inception in 2010, the Arab Spring has evolved into a situation of violent conflict in many countries, leading to high levels of migration from the affected region. Given the social impact of the large number of individuals applying for asylum across Europe in 2015, it is important to study who these persons are in terms of their skills, motivations, and intentions. DiPAS (Displaced Persons in Austria Survey) aims to uncover the socio-demographic characteristics of the persons seeking refuge who arrived in Austria in 2015, mainly originating from Syria, Iraq and Afghanistan. Particular focus is on human capital, attitudes and values. This survey, the first of its kind in Austria and possibly in Europe, was carried out among adult displaced persons, mostly residing in Vienna, yielding 514 completed interviews. Information gathered on spouses and children allows for the analysis of 972 persons living in Austria, and of further 419 partners and children abroad. Results indicate that the surveyed population comprised mainly young families with children, particularly those coming from Syria and Iraq. Their educational level is high compared with the average level in their country of origin. A vast majority of respondents are Muslims, rating their religiosity at medium levels. Judging from stated attitudes towards gender equity, interviewed men seem to have more liberal attitudes than their compatriots. The majority of respondents do not intend to return to their home countries, mostly because of the perception of permanent threat. DiPAS provides data for political decision-making and the on-going societal dialogue. Its findings can help to inform assessments about the integration potential of the displaced population into the host society. In addition, the applied methodological technique and experiences during the fieldwork provide valuable insights on sampling asylum seekers and refugees in the current European context.

## Introduction

Since its start in 2010, the Arab Spring has evolved into a situation of civil wars in many countries in the Middle East and North Africa region, most notably in Libya, Iraq, Yemen, and Syria, with the whole region now appearing to be unstable. The turmoil has led to waves of people seeking refuge, in a way unprecedented since World War II. While most of the displaced persons fled within their home country (7 million in Syria) or to neighbouring countries, e.g. from Syria to Jordan, Lebanon, and Turkey (4 million), some have been making their way to Europe [1], amounting to more than 1 million by the end of December 2015 [2]. Refuge-seeking persons from Middle Eastern countries have been joined by those of other nationalities, such as Eritreans and Afghans, who have acted upon the de facto liberalisation of policies regarding refugee flows in many European countries.

Austria–a country in Central Europe–traditionally receives high numbers of asylum seekers, due to its geographical location, the historical legacy of the Habsburg Empire and the historical political turmoil in the neighbouring Eastern European countries (e.g. Hungary, Former Czechoslovakia, Former Yugoslavia) [3–6]. During recent decades, Austria was considered as “a preferable refuge and a friendly host” ([4], p. 529), even though its policies regarding asylum and access to the labour market have always been quite restrictive [7]. While in 2015 the vast majority of the persons seeking refuge in Europe aimed to apply for asylum in Germany (which received almost half a million asylum applications in 2015 [8]), a substantial share also came to Austria in that year. In total, 88,098 individuals applied for asylum in Austria in 2015 [9]. This number corresponds to about 1% of the Austrian population and almost 7% of all asylum applicants in the EU in 2015, which made it the 4^th^ biggest receiver of asylum seekers in that year [10]. The inflow of displaced persons was highest in summer and autumn, with roughly 60,000 asylum applications filed between July and December (S1 File).

The citizenship of asylum seekers varies both temporally and regionally within European countries [1, 9, 11–14]. Most asylum applicants in Austria in 2015 were Syrians, Afghans and Iraqis (71%). The share of these three citizenships was large also in countries such as Germany, Sweden, Netherlands, Finland and Norway that witnessed heavy inflows of displaced persons in 2015 [9, 11, 13, 14].

The present study addresses an important research gap that stems from the general scarcity of quantitative data and studies on forced migration and displaced persons (e.g. [15]), along with a particularly acute lack of data about the recent arrivals of persons seeking refuge in Europe. This scarcity arises partly due to the difficulties in sampling forced migrants, owing mostly to the volatility of their status and their protection. Notable exceptions are surveys among Palestinian refugees in Lebanon [16] and among Syrian refugees in Turkey [17].

Displaced persons resulting from forced migration, according to the definition of the International Organization for Migration (IOM), leave their countries to escape man-made or natural situations that endanger their lives, freedom or livelihood. They encompass several categories: Refugees (persons who are recognized as such under the term of the 1951 Refugee Convention), subsidiary protected persons (who are granted temporally restricted humanitarian protection), asylum seekers (persons displaced outside of their national borders who have formally applied for international protection but whose claim has not yet been determined by the receiving state) and internally displaced persons (who are displaced within their own nation states).

Both the needs of displaced persons and the challenges of the receiving societies have been studied, often using interdisciplinary approaches [18–23]. Several themes are recurrent in the research on forced migration, such as: Vulnerable groups (e.g. women and children), individual differences in cultural identity, opportunities of displaced persons to fully participate in their host communities, inclusion in the host society, politics and policies of reception, as well as intention to return home [1, 4, 7, 15, 18, 24–29].

These studies employ various methodological approaches, both qualitative and quantitative. Whereas researchers typically apply methods used within their academic discipline [30], sampling techniques used in qualitative research (like snowballing) have been suggested as an appropriate methodology in quantitative research when working with difficult-to-access populations [31, 32]. Typically, research conducted on refugees is within one community or within one locality and cooperation with NGOs is frequently necessary [31, 33–36].

A recent review of surveys of refugee populations concludes that “[t]he paucity of statistics on refugees and related categories derived from sample surveys, [sic] is striking” ([37], p. 2), especially when one considers the last fifteen years. There are few studies focusing on the characteristics of displaced persons coming to Europe in recent years. A study by UNHCR [35] on Syrians arriving in Greece between April and September 2015 and seeking refuge (either in Greece or other European countries) revealed high levels of education (86% with a secondary or university education level).

Given the heavy inflows, the economic consequences for the receiving countries are increasingly studied in the European context [38]. Regarding asylum seekers’ potential contributions to the host country’s economy and labour market, scholars examined macroeconomic effects of the refugee influx for the EU [23] as well as for Turkey [39]. An OECD study discussed the difficulties faced by refugees and persons who have been granted some sort of protection when entering the host society’s labour market and educational system [27]. The authors conclude that integration efforts need to be customised because refugee populations are increasingly diverse in terms of family context, education, professional qualifications, and nationalities. This has been also addressed recently on national levels [38, 40, 41].

Further studies in the European context focused on the choice of country of refuge and return to the home country. A study carried out in Norway explored the dynamics of asylum movements and sought to answer the question of how refugees choose asylum destinations [42]. Some other studies address refugees’ potential to return to their countries of origin after the situation of crisis has stabilised [43, 44].

The latest wave of displaced persons into Europe has resulted in a new migration situation. In order to learn more about the displaced persons’ characteristics and also to identify their integration potential, several surveys are currently in preparation. A survey on refugees in Germany will be carried out in 2016, headed by the German Institute for Economic Research in conjunction with the German Socio-Economic Panel (SOEP), which will focus on the living conditions of refugees in Germany [45]. Recently the Max Planck Institute in Göttingen announced the launch of a study on the diversity of asylum seekers in Germany [46]. Moreover, data collection in Italy and Greece has been commissioned by the World Bank, in parallel with work in Turkey, Jordan and Lebanon. Due to the varied backgrounds and structure of asylum seekers in individual countries, all of these studies are highly relevant to address country-specific challenges, with the current study offering the first insights for Austria.

This survey, called DiPAS (Displaced Persons in Austria Survey) explicitly focused on displaced persons with Syrian, Iraqi and Afghan citizenship due to the reasons stated above. The fieldwork was carried out in November and December 2015, aiming to uncover the socio-demographic characteristics of the persons seeking refuge who arrived in Austria in 2015. Given the societal relevance of the latest migration flows in the EU, it is of the utmost importance to not only determine how many individuals are seeking asylum, but also to investigate who these individuals are by studying their socio-economic characteristics. In other words, not simply counting heads but also revealing what these heads contain in terms of aspirations, values, identities, and skills, and what they can offer in terms of human capital and integration potential for the host country’s society, in turn to better inform policymakers. At the individual level, education is found to be the most crucial factor in enhancing one’s capability to determine one’s own life, for example lifestyle, norms, behaviours, and attitudes, but it also relates to better skills and labour market opportunities, which subsequently leads to observable positive macroeconomic effects in the host country [47].

## Material and Methods

Given the complexity of surveys on displaced persons, the preparation, sampling and data collection for DiPAS are described in detail. Within the literature on displaced persons, scholars are increasingly addressing ethical and methodological aspects of the collection of data [30, 48, 49]. On top of the usual difficulties in surveying any trait in any particular population, displaced persons are in a situation of emergency and their management by local governmental and non-governmental organizations, very often on an ad hoc basis, renders sampling difficult [25, 32, 50, 51].

When considering central methodological issues that emerge when carrying out surveys with refugees, Bloch [49] stresses three main factors, which are true for displaced persons in general: “First, refugees are for the purposes of research a hidden group. Secondly refugees are not identifiable in official statistics. Thirdly, government ministries will not provide researchers with information to locate refugees for reasons of confidentiality.” ([49], p. 139). Representativeness and possible biases have to be addressed within the context of any survey and scholars have pointed out that sampling respondents is one of the main challenges faced when carrying out surveys with refugees [30, 48]. Some go as far as concluding that “representativity is an unachievable ideal in survey research on refugee populations” ([52], p. 110). Due to the paucity of data on refugees and asylum seekers from which to sample, surveys are usually based on non-probability techniques and mostly rely on access to refugees through community-based organizations or larger refugee NGOs [30]. It is argued that research on displaced persons always involves a degree of compromise [36, 53].

### 2.1 Study Design

Scholars underline that prior knowledge of the target group is central, especially when no sampling frames are available [30, 49]. Accordingly, in-depth exploratory work on allocation of displaced persons to residences in Austria in the context of the large inflows in 2015 was carried out prior to the actual DiPAS survey. Basic data on the number of asylum applications are typically collected by official institutions and government-related agencies. Monthly statistics show that the autumn of 2015 was the season with the highest numbers of asylum applications (S1 File). Due to the high numbers of refuge-seeking persons in 2015 in Austria, large emergency quarters were established to accommodate them, as well as to provide basic assistance to those transiting through Austria on their journey from the Hungarian and Slovenian borders to other destinations (mainly Germany and the Nordic countries). Transiting displaced persons were partly accommodated over a short period of time in special transit quarters and were not captured in the current survey by design.

The assignment to emergency quarters by the Austrian Federal Ministry of the Interior was random, especially in terms of individual characteristics including citizenship and human capital. A large share of the asylum seekers was first accommodated in Vienna, Austria’s capital and largest city by far (located in the East of the country close to the Hungarian border, from where most asylum seekers were arriving), before being assigned to more permanent housing facilities all over the country, including rural areas. In Vienna, the high concentration of asylum seekers in emergency quarters is indicated by the fact that by the end of November 2015, when the fieldwork was conducted, one-third of all displaced persons receiving basic assistance were residing in such accommodations. (In Austria, displaced persons receive basic assistance while their asylum application is pending, as well as for a transition period of four months following the approval of their application.)

For reasons of financial and logistical feasibility, our strategy was to focus on newly arrived displaced persons residing in these large emergency quarters. The survey was conducted in seven NGO-run refugee housing facilities in and around Vienna, among them four large emergency quarters and three smaller locations (in Austria, only a small number of care centres for displaced persons are managed directly by the federal state–the vast majority are operated by NGOs on behalf of the federal provinces.) Within each accommodation, interviewers approached individuals to ask for participation. Additionally, in the large emergency quarters we benefitted from indirect snowball effects, as those who gave an interview approached others within their accommodation and told them about the survey. The fieldwork was carried out during November and December 2015. Approval was obtained from the Ethical Committee of the Austrian Academy of Sciences. Participants provided their verbal informed consent to participate in the study; the interviewer read out the introductory text to the questionnaire and the participant verbally agreed to participate. Written consent was not obtained to ensure anonymity of the participants. We did not document participant consent, as only the participants giving their consent were interviewed. The ethics committee approved our procedure.

### 2.2 Data Collection

The questionnaire was based on the LFS (Labour Force Survey), WVS (World Value Survey), SHARE (Survey of Health, Ageing and Retirement in Europe) and GGS (Generations and Gender Survey) [54–56]. Moreover, specific questions to investigate potential inclusion in the labour market were added. The questionnaire (S2 File; Arabic and Farsi/Dari questionnaires available upon request) was organised around five main themes:

1. Demography: Age, gender, country of origin, ethnicity, marital status, former place of residence;
2. Human capital: Highest educational attainment (ISCED97 classification), type of schooling, occupational trainings, language competence;
3. Employment: Former participation in the labour market, type of employment (NACE and ISCO classification), number of hours worked;
4. Health: Self-perceived health, grip strength, limitation in activities of daily living;
5. Attitudes and values: Religion, democracy, gender equity, division of household work. Data were collected using computer-assisted personal interviewing (CAPI) techniques.

### 2.3 Recruitment and Training of Interviewers

The interviews were carried out by volunteers and students. Interview languages were Arabic, Farsi/Dari and English, to ensure that the majority of respondents could be addressed in their native language or a lingua franca. This strategy aimed to avoid a bias towards more highly educated respondents that would be the result of only offering the questionnaire in English, or requiring basic literacy, as is the case with paper and pencil questionnaires. Interviews in Arabic and Farsi/Dari were carried out by native speakers residing in Austria, many of whom had a refugee background. Although the participation of refugees in data collection and research has to be critically assessed from ethical and methodological perspectives [57], the inclusion of refugees as bicultural aides in the development of the survey and as interviewers turned out to be crucial. All interviewers received extensive training, including intercultural competence training focusing on diverging cultural backgrounds between the interviewers and respondents. After the fieldwork, interviewers were offered a psychological supervision meeting by a clinical psychologist.

### 2.4 Pre-Tests and Adaption of Questionnaire

Prior to the fieldwork, an intensive pre-test phase with 52 interviews was conducted, which consisted of multiple small-scale pre-tests and statistically analysed interviews.

This phase was crucial for identifying questions that would prove too sensitive, controversial or otherwise difficult and could result in the termination of the interview by the interviewee. Both in the development of the questionnaire and its adaptation based on pre-tests, aspects of access, trust, vulnerability and fear were considered [30, 58]. Additionally, the pre-test phase was necessary for technical tests of interview equipment, and to identify the legal and actual feasibility of conducting interviews in different interview situations and facilities. Insights gained during the pilot phase are potentially valuable to other social science disciplines and can be further analysed and triangulated with other qualitative and quantitative research studies on displaced persons.

### 2.5 Fieldwork Observations

The use of native Arabic- and Farsi/Dari-speaking interviewers and interpreters helped to establish trust and ensure that the sections explaining the survey’s purpose were truly understood (for research on the participation of refugees in the research process we refer to [57]). Respondents were repeatedly reassured of the purely scientific purpose of DiPAS in the course of each interview. Interviewers systematically emphasised that the survey was part of an independent academic research project, not related to government institutions, and would not affect respondents’ asylum application or status (S2 File). Furthermore information sheets in English, Arabic, and Farsi/Dari were distributed at all facilities prior to the actual interview dates, to introduce the team of researchers, and to explain the purpose of the survey. Taking all these measures into account, the DiPAS team judges that respondents were made sufficiently aware of the purpose of their participation in the survey, a particularly pertinent question for studies in which interviewers and respondents have diverging national and cultural backgrounds [42].

According to the UNHCR, “[i]n research involving interviewing refugees it must be borne in mind that ethical considerations are relevant. Not only may experiences of trauma and insecurity have characterised an individual refugee’s flight and journey, but such experiences often continue into the settlement context and may influence the individual’s ability and desire to integrate. These experiences may also affect refugees’ willingness and ability to participate in research” ([25], p. 23). Others have raised the issue of trust and mistrust both of and towards refugees [53, 58, 59], as the experiences of displaced persons in their country of origin and–if externally displaced–on their way to the country where seeking protection, might create their mistrust at various levels. Throughout the project, these restrictions and special conditions for interviewing recent refugees in Austria were taken into consideration and, if applicable, addressed in all stages of survey design, implementation, and analysis.

### 2.6 Sample Size

In total, 528 complete interviews were conducted, with the average time for one interview being 20 minutes. Another 22 interviews were started, but aborted by the respondents for various reasons (e.g. one person did not want to speak about the family, some had to leave for language courses). Almost two-thirds of the interviews were mainly carried out in Arabic, 20% in Farsi/Dari, and 11% in English. In total, 47% of the interviews were completely conducted by a native speaking interviewer, 32% were mainly interpreted by native speakers, 5% were partially interpreted by native speakers and 16% were supported by written translations only. Of the 528 interviews, 37% were conducted by male and 63% by female interviewers. A substantial number (33%) of those interviews carried out by female interviewers were conducted in English and assisted by male Arabic-, Farsi- or Pashto-speaking bicultural aids.

Restricting the sample to adult persons arriving in 2015 leads to a final sample of 514 interviews. Among these, 81% arrived in Austria between September and November 2015, showing that the sample captures particularly those who arrived in large numbers in autumn 2015. The final sample comprises 38% of respondents with Iraqi citizenship, 36% with Syrian, 16% with Afghan, and 10% with other citizenship (S1 Table). Therefore, almost three in four respondents were Iraqi or Syrian citizens. The 514 records include information on partners and children so that in total 1,391 individuals are captured in the survey, 972 of them living in Austria at the time of the interview and 419 living abroad (S2 Table). We are aware that information on family members might include bias in reporting.

### 2.7 Sample Validation

We calculated the participation rates in the different locations of interviews. To validate the DiPAS sample for representativeness, we compared it to several existing statistics on the population of refugees/asylum seekers in Austria and Vienna, in terms of numbers, age, and citizenship.

Participation rates in the different housing locations were calculated for the locations where the numbers of adult residents were provided. In the largest emergency quarters participation rates amount to 28% and 44% respectively, and in two of the smaller quarters they were as high as 64% and 77%. Unfortunately, participation rates cannot be calculated for all seven locations due to the incompleteness of provided lists or data confidentiality issues in some locations.

The DiPAS sample is first compared with the number of asylum seekers in Austria. The size of the DiPAS sample corresponds to 1.2% of persons seeking asylum in Austria in 2015. Since all of the respondents arrived before December 2015, the size of the sample increases to 1.4% of those arriving in the first eleven months. Available aggregated data allow a differentiation by citizenship and broad age groups. A comparison of all asylum seekers in Austria in 2015 (excluding unaccompanied minors) with the 972 asylum-seeking individuals living in Austria captured in the sample reveals an almost identical distribution in terms of age.

Further specification by citizenship shows that among Afghans, the proportion of children included in the DiPAS sample is higher than in the general refugee population (41% versus 35%), which might be explained by the fact that the comparably small group of Afghan respondents includes several persons with particularly large families (4 to 6 children). Among Syrian asylum seekers captured in the DiPAS sample, the share of children below the age of 18 is smaller (25% versus 34%), whereas more Syrians were aged 18 to 45 years (65% versus 59%) or 46 to 60 years (9% versus 6%). Deviations in the distribution by large age groups among Iraqis are very small and for those with other citizenships the distribution in the DiPAS sample is almost identical to the one for the whole of Austria. To summarise, the comparison with the total of roughly 80,000 asylum seekers (not including unaccompanied minors) by age group and citizenship indicated no evidence of a substantial bias in the DiPAS sample.

Next, our sample was compared with available official data on individuals residing in emergency quarters and receiving basic assistance in Vienna. The sample (including respondents, their spouse, and children if living in Austria) represents 15% of individuals residing in emergency quarters in Vienna at the time of the fieldwork. The proportion is high for Syrian and Iraqis (19% and 28% respectively), meaning that the current survey captures a substantial share of Iraqi and Syrian asylum seekers living in Vienna. However, the proportions of Afghans and other citizenships are low. Given this low share on the one hand, and the overall low number of Afghans in the DiPAS sample on the other hand, the results for Afghan asylum seekers should be considered with caution. Nevertheless, they provide valuable insights into this population.

## Results

We explore below the themes covered by the DiPAS questionnaire in terms of the demographic characteristics, educational attainment, language knowledge, skills and competences, health, religion and attitudes.

### 3.1 Demography

As shown in S1 Table, the majority of respondents were men (82%). More than 50% of men were below age 30, while the proportion was around 36% for women. Conversely, there are more women than men above the age of 40 years (27% versus 17%). Three in four interviewees were seeking asylum in Austria, while for some the country of application was still unclear (16%). Given that most respondents had just arrived, only a few held refugee status at the time of the interview and were thus holding a Convention Travel Document. The survey captured the large inflows in fall 2015 as seven in ten of the interviewed displaced persons arrived in Austria in September or October 2015.

Thirty-eight percent of respondents had Iraqi citizenship, 36% had Syrian, 16% had Afghan, and 10% had other citizenship (S1 Table). Iraqi respondents came mainly from Baghdad, Nineveh, and Basra, Syrians mainly from the governorate of Aleppo, Damascus and Homs.

Before fleeing to Austria, eight in ten respondents were living in their own home or in their family’s home, while only two in ten were living in rented accommodation. Property ownership was common among Syrian (87%) and Iraqi (76%) respondents, and to a somewhat lesser extent among Afghans (67%). Almost all respondents travelled to Europe through Turkey. Roughly one in four estimated their per-person travel costs to Austria to be less than US$2,000, and about one in four paid US$2,000 to US$2,999. About 20% paid US$3,000 to US$3,999, and 30% mentioned costs of US$4,000 or more per person (S1 Table). If we consider that per capita income per year amounted to about US$300 in Syria in 2011, such costs correspond to an average income of ten years in pre-war times in Syria [60]. Families travelling together had to pay a multiple thereof. Given the fact that exchange rates have seen drastic changes between 2011 and 2015, the average costs of travel found by DiPAS (in 2015) indicate a much higher financial burden in real terms.

Taking into consideration the interviewed displaced persons, their spouses, and children living in Austria leads to a number of 972 individuals included in the survey (S2 Table). In the overall sample of individuals living in Austria, two-thirds were aged 16 to 45 years, about one in four were children below the age of 16, and a small share (7%) were over the age of 45. Among Afghans, the share of children below the age of 16 was larger (37%), Syrians were more often over the age of 45, although this share remains below 10% in the current survey.

**Fig 1:**
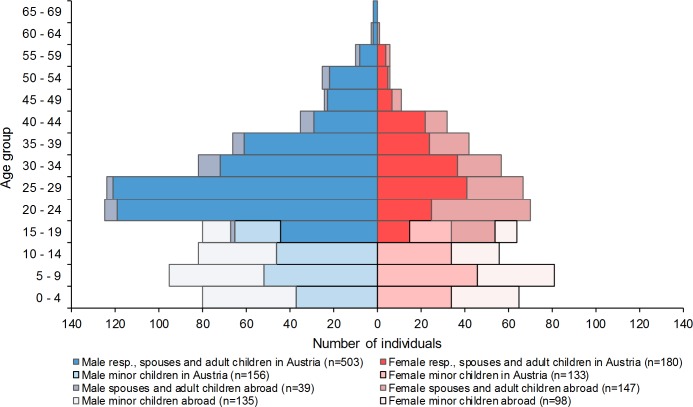
Family structure of displaced persons captured in the survey, by gender and age. Source: DiPAS, n = 1,391 individuals captured in the survey.

Information on the family members still abroad is important in order to assess the number and characteristics of individuals who may later join the current asylum seekers through family unification if/after they have been granted asylum in Austria. In addition to the 972 individuals already living in Austria, a further 419 close family members (i.e. spouses or children) were abroad at the time of the interview. Further specification shows that the population eligible for family reunification (373 individuals) consists of children under the age of 18 and spouses. When this number is related to the 972 adults already living in Austria, the estimated potential for family reunification can be given as 38 individuals per 100 asylum seekers in our sample (14 spouses and 24 minor children, see S2 Table). Children over the age of 18 represent a smaller group and are furthermore not eligible for formal family reunion. An age pyramid for displaced persons captured in DiPAS living in Austria and abroad is shown below (Fig 1).

The family status of the individuals captured in the survey who were living in Austria at the time of the interview was as follows: 39% were married, 3% divorced or widowed, 23% were single, and further 5% were adult children travelling with their parent(s), whereas 30% were minors below the age of 18. The composition varied to some extent per subgroup, with the proportion of married individuals being highest among Syrians (42%) and lowest among Iraqis (36%). Men in their twenties represented the largest group of unmarried individuals (S1 Fig).

### 3.2 Educational Attainment

The potential for inclusion in the labour market of the refugee population is comprised of two main components: Their education and their professional skills [61]. The positive selection of migrants with greater education and skills has been widely documented and although it is not universal, the theory that migrants tend to have higher than average skills compared to the general population of their country of origin tends to hold in the study of migration streams to EU and OECD countries [62]. While refugees and asylum seekers differ from economic migrants, one might nevertheless expect that those individuals crossing multiple borders by paying their passage to smugglers would usually come from the medium economic strata of the population, and therefore have had access to education. The poorest, and presumably least educated, would not be able to afford to make the journey and would therefore be displaced within their country of origin or to neighbouring countries instead. Also, the less well-educated might be more inclined to join the armed forces than those with more education who have access to other opportunities, including draft dodging, which has become prevalent in Syria in the years since the beginning of the civil war.

In this section, the analysis concentrates on the educational attainment of the displaced persons in the DiPAS sample (including the adult children and the spouses of respondents who are already in Austria). Furthermore, the educational level of the persons in DiPAS is compared to that of the general population in the country of origin (Syria, Iraq, and Afghanistan), and in the country of destination (Austria), to gain important information on the selectiveness and comparability of the sample. The analysis does not control for the quality of education of the different populations and simply focuses on the quantity, which could form a potential caveat. However, preliminary analysis by the AMS (Public Employment Service Austria) [63] shows that refugees’ levels of competence and skills are largely in line with their levels of education and/or occupation, which would increase their potential for integration in the host country’s labour market.

**Fig 2:**
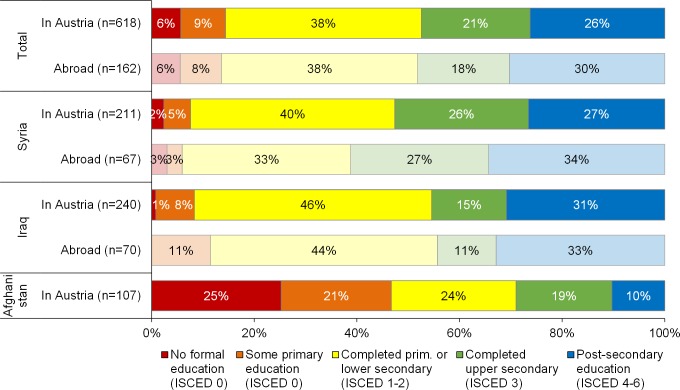
Educational attainment of respondents, spouses and adult children in Austria (saturated colours) and abroad (pale colours), 20–59 age group. Source: DiPAS. Remark: The count of Afghans living abroad is too low in the sample to be represented (n = 22).

The share of the respondents who have received no formal education at all, or just spent a few years in primary school without completing the final grade is very low in the sample. It is around 15% for all respondents (including spouses and adult children in Austria), but much lower among the Syrians (7%) and Iraqis (9%) than among the Afghans (25%) (Fig 2). Conversely, the large majority of displaced persons who arrived in Austria in 2015 have achieved at least compulsory education; 70% of the sample including the respondent’s spouse and adult children. (Compulsory education in Syria and Afghanistan consists of six years of primary education and three years of lower secondary education. In Iraq, only the completion of primary education is compulsory.) We did not find a statistically significant difference between men and women (not shown in the figure).

At the highest levels of the education scale, Fig 2 shows that 47% of the respondents in the sample have at least an upper secondary education and 26% have a higher education (post-secondary education, encompassing tertiary education e.g. bachelor degree as well as shorter post-secondary courses). The share that reported higher education is as high as 31% in Iraqi respondents and 27% in those from Syria. When distinguishing between college/bachelor’s and master’s degree, the overwhelming majority of the population with a post-secondary education hold a college/bachelor’s degree (87%). Some Syrian male respondents mentioned during the interview that they did not complete university studies to avoid recruitment in the Syrian army. Many young respondents expressed the wish to finish their studies in Austria (see also section 3.3). The difference in levels of education between the three main nationalities is pronounced: While 53% of the Syrians and 46% of the Iraqis have at least an upper secondary education, this share is below 30% among the Afghans.

The figures presented above do not change radically whether considering the sample of 618 adults present in Austria or the 780 persons including the population abroad. Although information on partners and children are proxies only and might be biased, looking at people left behind is important, since a large share of those might reach Austria through family reunification in the coming years (especially from Syria) once refugee claims are accepted. It is noticeable that the respondents who arrived from January to August 2015 were to some extent less well-educated than those who came after August: 37% of the early-2015-arrivers had an upper secondary or higher education, compared with 49% among those who arrived in the three months after August. Moreover, there were also more individuals with at most primary schooling among those who arrived from January to August (39% compared to 29% among those who arrived later).

**Fig 3:**
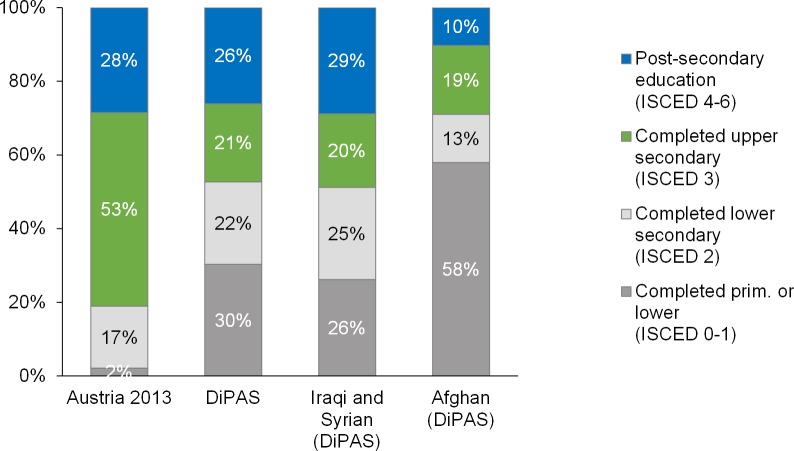
Educational attainment of the Austrian population and DiPAS respondents, spouses and adult children in Austria, age 20–59. Source: Register data for 2013, Statistics Austria and DiPAS.

The comparison to the Austrian working age population shows that the share of highly educated native residents is comparable with the share in the refugee population, in particular Syrian and Iraqi respondents (29% compared with 28% in the Austrian population) (Fig 3). Almost half of the respondents from Syria and Iraq have completed at least an upper secondary education. However, the total share of respondents in the sample who have completed an upper secondary education (21%) is less than half that of the Austrians (53%). Conversely, the share of respondents with at most primary education is much higher among the respondents from the DiPAS sample (30%) than among the Austrians (Fig 3).

Gender differentials in the levels of educational attainment reported are quite low. The difference is only clearly visible for those who have not received any formal education, where the share for women is higher than that for men: 10% and 4%, respectively. This finding is in line with most studies about the gender gap.

The share of persons with at most some primary education is higher among the older age group in both men and women, and the share of those with secondary or higher education is lower for both genders. Those aged 20–29 years (the most numerous 10-year age group in the sample) are more educated than those aged 30–59 years: 55% of those in the 20–29 age group have an upper secondary education or more, compared with 41% in the 30–59 age group. The general pattern stays the same but the differences are pronounced between the Syrian and Iraqi respondents and other citizens (mostly Afghans).

Our conclusions on the general levels of educational attainment of the respondents appears to corroborate the findings of the survey on the competences of 898 refugees implemented between August and December by the AMS [63]. The levels of education observed at the level of each country of citizenship is very similar: 55% of the Syrians have attained an upper secondary or higher education according to the AMS, and 53% according to DiPAS. For the Iraqis, the corresponding shares are 55% and 46%, and for the Afghans, 24% and 29%. It is worth noting that the main difference is among the samples of Iraqi citizens, where the AMS sample is more limited (40 persons compared to 240 persons in the DiPAS sample). This difference stems from the gender differentials, where Syrian women interviewed by the AMS tend to have attained higher levels of education compared to men in the AMS sample, and also compared to both men and women in the DiPAS sample.

When capturing education in DiPAS, we did not ask for certificates as it would have complicated or disrupted the interview. As respondents had only recently arrived in Austria, they might not have had time for formal translation or accreditation by Austrian authorities. Additionally, they might not have been able to provide certificates at all, having them left behind or lost them during their journey to Austria. The competence checks conducted by the AMS to some extent try to verify missing certificates [64]. For similar reasons, the Austrian government recently passed a law on the recognition and assessment of qualifications to support immigrants in receiving quicker accreditation of foreign degrees and documents acquired abroad (see also [64]).

**Fig 4:**
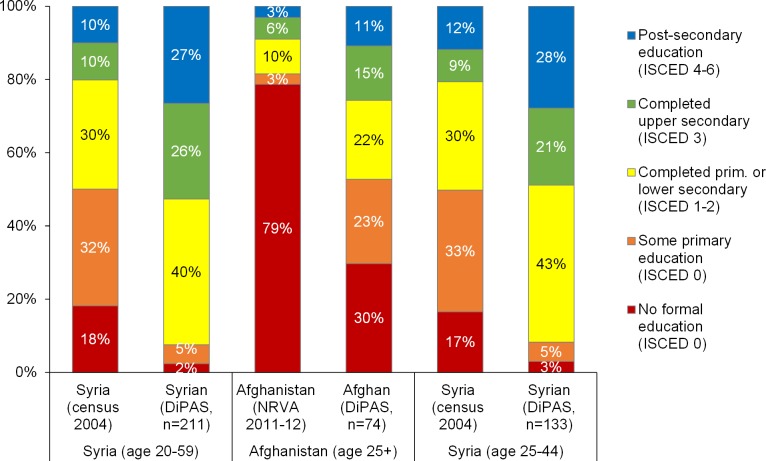
Educational attainment of the general population in the country of citizenship and DiPAS respondents, spouses and adult children in Austria, aged 20+ years. Sources: DiPAS, Central Bureau of Statistics (2004) for Syria, Central Statistics Organisation [65] for Afghanistan. Notes: No recent representative survey is available for the Iraqi population. Age ranges reflect availability of data.

As previously mentioned, one might expect that displaced persons would be more educated than the general population in their country of origin. The comparison between the two groups is not easy due to data availability issues. However, by looking at the high share of refugees with post-secondary education, it becomes clear that the forced migrants differ greatly from the general population. The simple comparison shown in Fig 4 illustrates that the interviewed Syrian and Afghan displaced persons are much more highly educated than the general population. For Syrians, this comparison is based on the last reliable data from the 2004 census. As individuals in the country of origin are on average older than persons included in DiPAS, we restricted–for Syria–the comparison to the age group 25–45 and found similar results (Fig 4, right part).

Although Afghan displaced persons have much lower education levels compared to Syrians and Iraqis, they are much more highly educated than the adults of working age in Afghanistan. The lower general education level of the Afghans stems from the severe conditions that affected the education system in Afghanistan for many decades, due to war, for example after the Soviet invasion in 1979, and also due to regimes that oppose schooling such as the Taliban regime in the late 1990s.

As shown earlier, Iraqi respondents have the highest levels of education, with as many as 31% post-secondary educated. This indicates that they are also a very strongly self-selected group and not representative of the general Iraqi population, which, according to the latest Multiple Indicator Cluster Survey (MICS) survey conducted by UNICEF [66], has a majority with primary education or less (51% among household heads and 57% among women).

Within a broader perspective of education and human capital, we briefly refer to language competence and knowledge of the language of the receiving country, as these certainly help the displaced individuals to adapt to their new setting and culture, and are important for integration into the society as well as in the labour market. It turns out that 52% of the persons interviewed in DiPAS spoke another language in addition to their mother tongue (54% of Syrian, 49% of Iraqi, and 41% of Afghan respondents spoke another language). As many as half of those who spoke another language spoke Turkish, Kurdish or another local language, while 37% spoke English and an additional 13% another European language, including German (2%). These numbers clearly show the need for language courses to be provided to forced migrants in Austria.

### 3.3 Employment

While access to the labour market is highly restricted for asylum seekers in Austria and other European countries (cf. [67]), refugees and people under subsidiary protection who enter the workforce, despite language barriers and frequent loss of qualification certificates and other documentation, have a higher likelihood of being overqualified for the positions they work in [25]. This has also recently been confirmed for the German labour market, which shares many of its characteristics with the Austrian one [40]. Further challenges include issues of poor mental and physical health and difficulties in the translation and transfer of educational and professional certificates [7, 21, 22, 68]. Hence, forced migrants typically face more difficulties when searching for a job in the host country than regular migrants [69–71]. Conversely, studies have shown that refugees are highly motivated to catch up with the success of other migrant groups, partly due to a lack of return options [72], and that they have more often been self-employed than the population in the host country, which suggests a pronounced entrepreneurial spirit among displaced persons [73].

Regarding the previous work experience of respondents, an overwhelming majority (72%) of refugees have already participated in the labour market in their home country at some point in the past. Since a lack of work experience in the host society has been shown to be one of the main reasons for refugees’ difficulties when searching for a job [74], extensive work experience in the country of origin must be evaluated as a positive starting condition. Perhaps unsurprisingly, we found a clear gender differentiation between men’s (90%) and women’s (42%) levels of previous work experience. Many women in the DiPAS sample had young children, which may offer one explanation of their lack of participation in paid-work activities, along with other explanations such as a scarcity of jobs, security challenges in conflict-ridden areas, and cultural preferences. However, 75% of women with a post-secondary education reported previous work experience, compared to 35% among those with primary or secondary education. Education levels did not make a large difference to job experience among men as they are generally expected to be the main breadwinners. Notably, male respondents’ female spouses who had not yet arrived in Austria had a considerably lower likelihood (30%) of having actively participated in the labour market than female respondents or spouses who were already in Austria (51%).

Looking at the actual occupational fields in which respondents had gained work experience, once again a clear gender bias was detected. Overall, there are two main areas of occupation dominant among female respondents: Education (26%) and “other service activities” (20%). This trend correlates well with the distribution of female labour market participants in Austria [75]. Furthermore, about 11% of female respondents had been working in “human health and social work activities”, a sector which is widely regarded as having considerable growth potential due to Europe’s aging population and the rising demand in care workers for the elderly. This distribution may cautiously be assessed as favourable for the Austrian labour market and civil society, although such integration successes also strongly depend on which region in the host society refugees will eventually be settled in [71], especially if one considers Austria’s pronounced urban-rural divide.

A stronger contrast between the Austrian and the DiPAS sample populations can be found among respondents indicating work experience in “professional, scientific and technical activities”, a branch which represents 6% of the Austrian female labour force and 4% of the male one [75]. In the DiPAS sample, however, 10% of female and male respondents with previous work experience indicated that they had previously worked in this field. Given the fact that many available occupations within this category typically require upper secondary to tertiary education, it remains to be seen how far language barriers and lack of qualification documentation (such as work certificates and educational degrees) affect both male and female refugees’ entry into this particular sector of the Austrian labour market (see also [64]). Recent national policy initiatives, such as professional mentoring for refugees and persons under subsidiary protection and support for companies who provide language courses for such employees [76], may help to address these difficulties.

**Fig 5:**
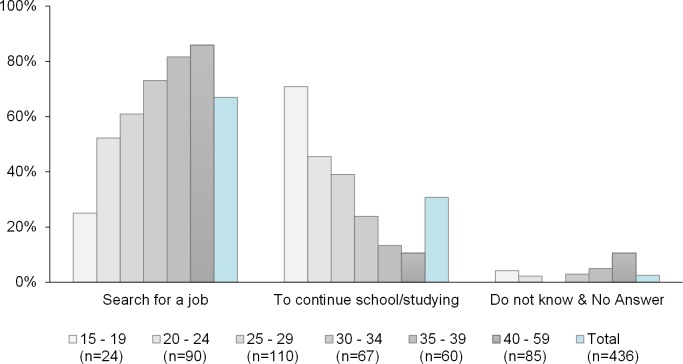
Intention to work and/or study after having received official asylum status in Austria, by age groups. Source: DiPAS, n = 437 individuals with previous labour market experience captured in the survey, living in Austria.

Besides questions on previous economic activity and occupation, respondents were asked about their plans, intentions and expectations for life in Austria, including possible plans for employment or further education (school, university, college). Fig 5 shows the distribution of all respondents with previous labour market experience, with the options “search for a job” and “continue school/studying” scoring the highest percentages (67% and 30% respectively).

Regarding the question on their future plans in Austria, the majority of respondents chose “search for a job”, but the option “continue school/studying” was predominant among younger respondents (Fig 5). In the relevant age groups of 15–19 years and 20–24 years, 71% and 46% respectively indicated that they wish to continue/complete their education in the host country after having been granted asylum or subsidiary protection. In comparison, only 25% and 52% respectively in these age groups intend to search for a job and enter the workforce straight away (Fig 5).

### 3.4 Health

The healthy immigrant theory [77, 78] states that there is a self-selection bias present among immigrants so that they are typically healthier than their compatriots who have not chosen to leave their country of origin, and also healthier than natives of the country into which they are trying to immigrate. Kohls [79] found that victims of civil war and asylum seekers fleeing violence and political instability will be less likely to fit this pattern because the motivations for fleeing violence and civil war are very different from those that prompt standard economic immigration, and are even likely to increase the number of unhealthy migrants within the population. Nevertheless, respondents included in the DiPAS sample appear to be in very good health: Most of them reported “good” or “very good” health (85%), no chronic health problems (84%) and not having been unable to work in the last six months due to a health related issues (83%).

In general, women report their health as being worse than that of men, with only 69% compared to 89% of men self-assessing their health as “good” or “very good”. This gender difference is observed not only in migrant populations but also in autochthonous populations [80]. In interpreting this gender difference it has to be taken into consideration that women in the DiPAS sample are on average almost three years older than men (the mean age is 33.3 years for women and 30.5 years for men), and that the small sample size could also affect the statistics. In comparison to Austrians [81] it would appear that displaced persons’ health status is polarised by gender, with males reporting better health (89% compared with 81% of Austrian males) and women reporting worse health (69% compared with 77% of Austrian females) than in the host country’s population.

The survey captures some basic background information on the respondents’ journey to Austria, including the question of whether individuals had spent some time in a nation other than their home country (e.g. refugee camps in Lebanon). There is also evidence of an inverse relationship between having moved away from one’s nation of birth and willingness to return home once ill. Those respondents who had not moved to another nation before leaving their homes as asylum seekers are more likely to be willing to consider returning home (although absolute numbers related to the earlier movers are here very low, suggesting caution in reading such a result). This result suggests that there could be a “homing instinct” among some individuals, but that those who have already moved away from home are less likely to wish to return to their country of origin.

### 3.5 Religion and Attitudes

Another important area of refugee integration about which little is known regards their values and attitudes. An often-voiced concern is that migrants and refugees, particularly Muslims, would reject Western values altogether and resist integration [82, 83]. Evidence from the World Values Survey (WVS) suggests the existence of a deep cultural divide between Islamic and Western societies and that the “true clash of civilisations” is not about democracy, but rather attitudes towards issues such as divorce, abortion, gender equality, and gay rights [84, 85], all of which can (but do not have to) be justified through a religious lens.

The religious identity of persons seeking refuge became part of the political and media discourse at a very early stage of the “refugee crisis” (e.g. [86, 87]). This is not surprising, since in most of the receiving countries, negative, mostly discriminatory and hostile attitudes toward Muslims prevail (e.g. [88] in France). While destination countries such as Austria often lag behind others in areas such as equal pay for equal work, or the gender distribution of childcare and work in the informal economy [89, 90], there is also broad evidence of “acculturation” among immigrants: Over time, immigrants tend to adapt their gender ideology to the standards of their country of residence, without necessarily ceasing to follow their own religious beliefs [91]. Norris and Inglehart ([92], p. 230) even go so far as to quantify this diminishing cultural distance, stating that “the basic values of Muslims living in Western societies fall roughly halfway between the dominant values prevailing within their countries of destination and origin.” It is unclear though whether this is equally true for people that did not migrate voluntarily but were forced to leave their countries of origin. To assess this “cultural distance”, as well as religiosity and how these two factors interact for the displaced persons arriving in Austria in 2015, respondents to DiPAS, who are evidently at the beginning of their journey towards integration, were asked to respond to questions about their attitudes toward gender equality, abortion, religious education in school, and religious intensity.

In DiPAS, respondents were asked about their own religious affiliation, but not about the religion of their spouse or children. As expected, the vast majority of the Iraqi, Syrian, and Afghan populations in the DiPAS sample are Muslim: 95% across the three nationalities and 88% in the overall sample including other nationalities (S1 Table). The difference is mostly due to the larger share of other religions, particularly Christianity, among Iranian respondents. This could be linked to the fact that migrants of the Iranian diaspora often seek to reject the rigorous Islamic identity imposed by the Iranian state [93]. Among Iraqis, Syrians, and Afghans only about 2–3% of were Christians. The share of Christians among the Syrian displaced persons is below the estimates of the actual size of Christian communities in Syria before the conflict. Potential reasons for this low number of Christians include that (1) Christians have tended to support the Bashar al-Assad regime and are less targeted by the attacks that are particularly aimed at densely Sunni-populated areas and (2) that Christians were more present in earlier waves, or went to other, non-EU countries in the Middle East or elsewhere, or (3) arrive through informal networks such as diaspora Syrian Christians already in Austria, and are thus not located in emergency quarters, but elsewhere. The share of respondents who declared no religion was negligible, especially among Iraqis and Syrians (0.5%).

The respondents were also asked to rate their religiosity, meaning the intensity of their religious practice and/or belief, on a scale from 1 (not religious at all) to 10 (very religious). Many found it problematic to quantify their religiosity and a large share of the respondents (40%) chose 5, the median value. It has to be mentioned that caution is needed with interpretation of the results on religiosity due to interviewer bias, as results differ for the respondents interviewed in their native language versus in English, with reported religiosity being lower among the latter group.

**Fig 6:**
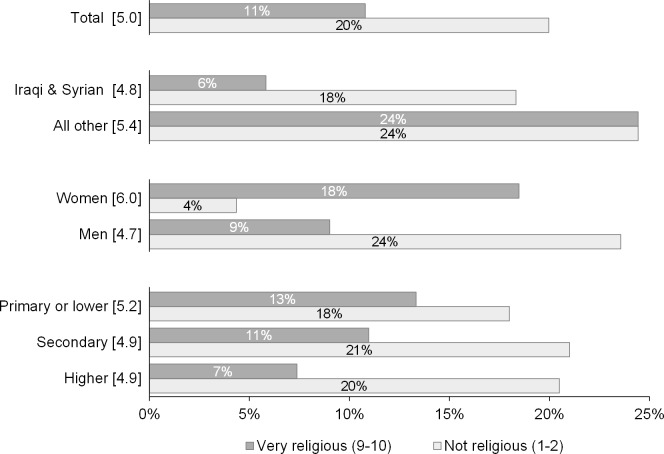
Percentage of respondents with very high or low values of self-assessed religiosity. **Source: Displaced Persons in Austria Survey (DiPAS).** Remark: Values in square brackets are the mean values reported by the given subgroup.

In accordance with the literature, women tend to be more religious compared to men [94] and the percentage of people identifying as very religious declines with increasing education [95]. When looking at the upper and the lower end of the distribution in Fig 6, the results indicate that more displaced persons report not being religious (20% who answered 1 or 2) than being very religious (11% who answered 9 or 10). Asked about their tolerance of other religions, a large majority of respondents to DiPAS (68%) would not mind if his/her child(ren) were taught in school about other religious traditions, as is the case in Austrian public schools.

The acculturation mentioned above in terms of attitudes about gender roles also has a strong effect on the ability of immigrant women to integrate into a host country’s labour market [96, 97], as immigrant groups from countries with less egalitarian gender roles tend to have lower female labour market participation and higher fertility rates [98]. These effects can persist into subsequent generations as gender roles are transmitted between generations and can lead to significant inter-ethnic gender wage gaps [99].

**Fig 7:**
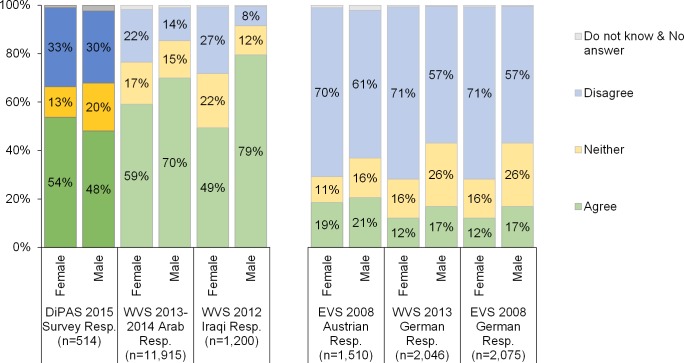
"When jobs are scarce, men should have more right to a job than women.” **Agreement in the Displaced Persons in Austria Survey (DiPAS) and in the European and World Values Survey.** Sources: WVS; own calculations; Displaced Persons in Austria Survey (DiPAS). Remark: “Arab respondents” include people from Algeria, Bahrain, Egypt, Jordan, Kuwait, Lebanon, Libya, Tunisia, and Yemen.

The first statement that respondents were asked to agree or disagree with was: “When jobs are scarce, men should have more right to a job than women”. Differences between the values subscribed to by men and women in DiPAS are rather small, with roughly 50% of both men and women responding in the affirmative (Fig 7, left chart). Women appear to be more decided and chose both “agree” and “disagree” more frequently, avoiding the neutral option, although the total number of female respondents is low. Nevertheless, comparison with the Iraqi WVS shows that the DiPAS results are very similar for female respondents, whereas men tend to be far less traditional in the DiPAS sample (Fig 7, left chart).

Responses to DiPAS more closely resemble the Iraqi results than the Austrian or German responses. In the two neighbouring German-speaking countries, which are not only in geographical proximity and share a common cultural history, but largely coincided in their initial political response to the refugee crisis, disagreement with the statement “When jobs are scarce, men should have more rights to a job than women” exceeded 60% already in 2008 among both men and women. However, men in the DiPAS sample disagree with the statement more often than Arab or Iraqi men in the WVS sample. On the one hand, this could be the continuation of an on-going trend towards a greater awareness of issues of gender equality in the Arab world, as suggested by the pooled results from nine countries in the region where the survey was conducted in 2013 and 2014 (Fig 7, left chart, middle columns). On the other hand, it could also mean that male adults who more strongly support women’s rights at home and at the work place tend to leave their country of origin more frequently than their more traditionally oriented peers. This perspective is supported by the evidence that conservative attitudes in general are associated with lower levels of education (results available on request).

Since the majority of the interviewers were female, whereas the interviewees were predominantly male, there is a risk of interviewer effects, particularly when they are asking questions about gender attitudes [100, 101]. However, when looking at the differences in response behaviour of the male respondents (only 35 women were interviewed by male interviewers), despite the small number of respondents, it appears that their behaviour hardly differs by the gender of the interviewer.

When asked about the role of women in the workplace, a vast majority of both women (85%) and men (68%) agreed that “Having a job is the best way for a woman to be an independent person”. This result stands in stark contrast to widespread expectations that people fleeing from predominantly Muslim countries would adhere to a traditional, exclusively-male-breadwinner model and is confirmed by responses to three other statements regarding the role of women in the workplace and society more broadly. While religion is an important influence on the attitudes included in DiPAS, particularly with regard to women working outside the family, even among the more religious male respondents predominantly gender-egalitarian views prevail (results available on request). Thus, the results based on DiPAS seem to dismiss the popular stereotype, described by Read [102] in the US context, of Arab women being Islamic traditionalists–veiled and secluded within the home.

DiPAS allows analysis not only of the respondents’ attitudes, but also their behaviours and decisions, by asking about household decision-making in everyday situations, such as shopping, childcare or the distribution of housework. Household chores are regarded traditionally as belonging to women, financial decisions to men. Among those currently or previously partnered, it is not necessarily the man who takes the decisions and the woman who is exclusively in charge of the household chores while not having any say in financial matters. In fact, decisions regarding the household finances were mostly handled jointly by the couple. It might be concluded that traditional gender roles are intact insofar as we found that very few (formerly) married men would take the larger share of the intrafamilial care or housework. However, a large percentage of female respondents confirmed that their husbands do at least help with these tasks.

### 3.6 Plans to Return Home

Considerations about the situation in the respondents’ home country are important for the nature and degree of integration of migrants, hence their permanent or casual assessment of political, security, socioeconomic, family, and kinship situations may play crucial roles in refugees’ decisions to stay in the host country or eventually return to their countries of origin [43, 44, 103]. These may have specific relevance to the victims of persecution, war refugees, displaced persons, or forced migrants in general. It is clear, however, that in a concrete decision-making process, both successful and failed (or deficient) integration may result in the decision to either stay or return, although with different probabilities.

The recently arrived displaced persons as interviewed in the DiPAS were evidently not in a position to decide about their long-term future. Having escaped the life-threatening situation at home, they were still waiting to be officially acknowledged as refugees by the Austrian authorities. It has to be noted that in the prevailing “welcome culture” of autumn 2015, respondents did not notice a hostile atmosphere within the host country. Hence, asking respondents about their inclination to stay or return could only be hypothetical. Nevertheless, the answers can be regarded as proxies or indicators for integration. The question used to test this intention reads as follows: “Would you consider to return to Syria/Iraq/your home country after the situation has stabilised?”, possible answers were (1) Yes, (2) No and (3) Don’t know.

Those replying “no” or “don’t know” were further asked about the reasons or conditions under which they could imagine returning. Two in three (67%) said that they would not be willing to return to their home country even after stabilisation. The remaining third was split between those who considered return (22%) and those who responded “don’t know” (11%) (S1 Table). The latter group had obviously not yet considered such an option. If the majority of those who are currently undecided end up deciding not to return, three out of four respondents would prefer to stay in Austria.

In a breakdown by citizenship, Syrian respondents showed the strongest ties with their home country: The proportion intending to return (32%) was 10 percentage points higher than the average (S1 Table). Syrians also had the highest share of undecided respondents (16%), but nonetheless the majority (52%) were not considering their return. Among Iraqis (77%) and Afghans (75%) as well as the other nationalities combined (69%) the proportion not considering returning to their home country was even higher. Interestingly, 42% of those reporting their most recent prior residence in their country of birth were willing to consider returning home compared to only 17% of those who reported their most recent prior residence in a country other than that of their country of birth. This echoes the DiPAS findings on health (section 3.4).

Both men and women were in favour of staying, with a slightly larger majority among men (68%) than among women (60%). A combination of worse health and slightly higher average age may be responsible for female respondents being more willing to return home than male respondents. Furthermore, there are no differences between single and married individuals, but in line with their worse self-perception of their health, divorced and widowed respondents were more willing to return home (29% and 40% respectively) than single and married respondents (24%). Finally, no consistent pattern according to the level of education was observed; in each education group those considering return were outnumbered. This is to some extent in contrast to a study that identified educational differences in voluntary return among displaced persons in Turkey, where better-educated individuals more often returned to their ancestral communities [103]. However, the latter focused on internally displaced Kurds and their effective return home after prolonged displacement, while the current study captured the intention of voluntary return of externally displaced persons. Their effective return to their home country can only be assessed with longitudinal data. Nevertheless, there is an observable trend for those with less education to be surer of their decision, as we observe the percentage of respondents who are uncertain falls with their level of education (results available on request).

The most important obstacle to return is to be found in the perception of permanent threat in the home country. Seven in ten agreed with the statement “I don’t think that the personal safety of my family and me would be guaranteed, even if the general situation stabilises”. The safety issue was given more weight than the political one, captured by “I don’t think that the general political situation will stabilise within the next years” (58% agreement). Economic reasons were rated less important, both at the macro level (“I don’t think that the economic situation will stabilise within the next years”) with 23% agreement and at the micro level (“I don’t think that I will be able to make a living in my home country, even if the situation stabilises”) with 16% agreement. Almost all denied financial reasons (“Even if I wanted to, I couldn’t afford to return”). Reasons of a more personal nature played also a very minor role: Only 8% found they would have “nowhere/no one to return to” and 7% that they had no more emotional attachment to their home country.

## Discussion

This project seeks to contribute to the evidence-based social debate on forced migrants, and their inclusion in society. The empirical basis established by our data is intended to assist national efforts in appraising refugees’ and asylum seekers’ potential for integration in the host society, particularly as concerns respondents’ general demographics, educational levels, and professional experience. Rather than merely “counting heads”, we aimed to uncover what these heads can offer in terms of human capital for the labour market of the host country and society at large.

The DiPAS project proves to be innovative in several aspects. First and foremost, it is the first survey of its kind focusing on the recent arrivals of Syrian, Iraqi, and Afghan refuge-seeking persons in Austria, and to our knowledge in Europe. Second, the focus of the DiPAS study, i.e. an informed assessment of the human capital and family context of displaced persons, is of particular significance in demographic research. Finally, the survey allowed the team members to carry out cutting-edge research and thus obtain unique expertise in a research field that will gain increasing relevance in the years to come. We hope that the pioneering DiPAS study will provide valuable input on further fields of inquiry, the design of suitable questionnaires, practical and ethical guidance for dealing with respondents with a refugee background, and useful general information on fieldwork management to prove helpful for similar surveys in other European countries affected by the large inflows of refuge-seeking persons in the last year.

The main findings of DiPAS concern human capital, family structure, value systems, and return intentions of the newly arrived refuge-seeking persons in Eastern Austria, particularly in its capital Vienna. In particular, Syrian and Iraqi respondents who have arrived since summer 2015 display consistently high levels of education, which partly refutes commonly-held public assumptions about asylum seekers’ and refugees’ low or non-existent education and alleged illiteracy. The share of respondents with no or minimal formal education (a few years in primary school) has proven to be very low in the DiPAS sample, around 15% for all respondents, and higher among Afghans (25%) while very low among Syrians and Iraqis (7–9%). Our findings show that asylum seekers of different origins have different characteristics and even those coming from less developed countries, such as Afghans, are positively selected in comparison to the general population in their countries of origin. While 53% of the Syrians and 46% of the Iraqis have at least an upper secondary education, this share is below 30% among the Afghans. This discrepancy is primarily linked to the historical legacy of the countries of origin. Our findings on respondents’ levels of educational attainment hence corroborate the results of the survey on the competences of 898 refugees by the AMS (Public Employment Service Austria) (AMS 2016).

Secondly, the analysis of displaced persons’ family structure yielded valuable results. A majority of respondents arrived with their nuclear family, especially in the Syrian national group. The greatest possibility for reunification lies with under-aged children and with mothers who may decide to join their family members who have already emigrated: Our findings show that the potential for family reunification (373 individuals) consists of children under the age of 18 (62%) and spouses (38%). Children over the age of 18 represent a smaller group and are not eligible for formal family reunion. These findings ought to be taken into consideration for national and international policymaking.

Thirdly, our findings on potential labour market integration of asylum seekers can be cautiously assessed as promising for the host society: An overwhelming majority (72%) of respondents have already participated in the labour market in their home country at some point in the past. The intention to participate in the host society’s labour market is equally high: 67% of all respondents intend to “search for a job” after having been granted official asylum status (refugee status under the 1951 Refugee Convention) in Austria. Among younger respondents, the option “continue school/studying” was predominant: In the relevant age groups of 15–19 years and 20–24 years, 71% and 46% respectively indicated that they wish to continue/complete their education in the host country. These findings corroborate a recent study by OECD suggesting ways to support the lasting integration of immigrants [27].

Findings on the attitudes and values of the newly-arrived persons seeking refuge, especially responses about religiosity and the (professional) role of women, may contribute to the on-going public debate about the entwinement of gender equity with religious attitudes (for example, following the recent events on New Year’s Eve 2015/16 in Cologne, Germany). Four questions in the survey assessed the prevalence of traditional gender-role ideologies (see items Q59, Q61, Q62, Q63 in the questionnaire). Against popular expectation, these were dismissed by a majority of both male and female respondents. While religion is an important influence on the attitudes tested, results show that even among the more religious male respondents, predominantly gender-egalitarian views prevail. When asked to rate how religious they consider themselves, the share of respondents reporting not being religious (20%) far exceeded the very religious (11%). In accordance with earlier findings, female respondents tended to consider themselves more religious (average score 6.0) than men (average score 4.7). The percentage of people identifying as very religious declines with increasing education.

Finally, respondents’ return intentions may provide valuable insights into expected long-term developments of the Syrian, Iraqi and Afghan refugee population in Austria. Three out of four DiPAS respondents prefer to stay in the host country, even after the situation in their home country stabilizes. Divided by citizenship, Syrian respondents were most willing to consider a return to their home country, with positive response rates at 10 percentage points higher than the average (S1 Table). In a breakdown by gender, men were slightly more in favour of staying than women (68% versus 60%). Permanent threat to livelihood in the home country was given as the most important obstacle to return, while economic reasons were rated as less important (ca. 70% to 23% on the macro and 16% on the micro level). Given these findings, long-term integration policies on the national level seem pertinent, especially as concerns refugees’ opportunities for earning a living in the host country.

Indeed, effective participation by refugees and asylum seekers in the labour market of the host society is widely considered to be a key integration indicator (cf. [25, 27]). According to a recent report published by the International Monetary Fund [23], rapid integration of asylum seekers and refugees into the workforce must be considered “key to reducing the net fiscal cost associated with the current inflow” of persecuted individuals from Syria, Iraq, Afghanistan, and other countries, and may “counter some of the adverse fiscal effects of population aging” ([23], p. 5) that heavily affect Western European countries. The main challenges, as well as good policy practices to support the lasting integration of immigrants and their children in the host countries, were recently summarised by the OECD [27].

This paper provides an overview of the first results of our survey and further, more detailed analyses for policy makers and integration experts may be generated. For example, one may wish to take the professional background of respondents, filtered through NACE and ISCO codes into account for assessing integration efforts and potentials. Moreover, additional in-depth analyses of our data, including multivariate methods, may answer the question of who the refuge-seeking persons arriving in large numbers in 2015 in Austria are, regarding their family situations, human capital, coupled partner analyses, health, plans for voluntary return, as well as their values and attitudes.

Several limitations have to be mentioned, especially in the context of representativeness. As outlined in the section on the study design, there is mutual consent among scholars in the field of forced migration that it is particularly difficult to generate representative samples of mobile populations. As a result, we had to accept some methodological compromises for the implementation of DiPAS, which however will not invalidate the findings or their overall relevance. The results for the Iraqi and Syrians are more robust than for those for the Afghans and other nationalities. Nevertheless, we do not expect any bias when comparing the different nationalities with the population in the country of origin. Official statistics on asylum seekers are available by gender and by citizenship, but unfortunately, data including both characteristics are not available. Moreover, statistics on gender include unaccompanied children, which were excluded in our study. For these reasons, we are not able to compare gender statistics to the population. To add, given the low number of female respondents, results for women are also less robust, and as a result, analyses including both men and women are biased towards male respondents. Where possible, we therefore carried out the analysis separately for men and women, and for the different nationalities, to avoid such biases.

Further aspects of representativeness relate to non-response and the characteristics of those not participating in surveys. Research on non-response bias in social surveys (capturing a country’s population and not focusing on specific groups like displaced persons) has identified various factors affecting survey participation, including education, urbanisation, age, parenthood status, migration background, economic situation, religiosity and health [104–108]. We are unfortunately unable to explore whether these factors affected participation in DiPAS. Even if official numbers on nationality, gender and age are collected by governmental bodies and partly made accessible to the general public and/or the scientific community, no official statistics are available on education and other aspects of human capital, not to mention personal dimensions like attitudes and values, which would allow us to more critically appraise the DiPAS data.

Country-specific studies are extremely important for informing national integration policies. In order to better understand the recent influx of displaced persons, comparisons with other countries may reveal further important findings. The currently on-going and planned initiatives for data collection in various European countries will allow valuable comparisons with the Austrian findings. Therein we regard the current survey and study as a small but important piece of a big puzzle which helps in gaining a clearer picture of persons seeking asylum in Europe in 2015, specifically in Austria.

## Supporting Information

S1 Data Data files. (ZIP)

S1 Fig Family status of individuals captured in the survey living in Austria, by gender and age. Source: DiPAS, n = 972 individuals captured in the survey, living in Austria.
(TIF)

S1 File Historical and comparative perspective on the flows of displaced persons in Austria. (DOCX)

S2 File English questionnaire. (PDF)

S1 Table Characteristics of respondents. Source: Displaced Persons in Austria Survey (DiPAS), n = 514 interviewed persons.
(DOCX)

S2 Table Individuals captured in the sample. Source: Displaced Persons in Austria Survey (DiPAS), n = 1,391 individuals captured in the survey.
(DOCX)

## References

[1] Fargues P. 2015: The year we mistook refugees for invaders Florence: Migration Policy Center; 2015.

[2] IOM. Mixed migration flows in the Mediterranean and beyond: Compilation of available data and information Geneva: International Organization for Migration; 2016.

[3] UNHCR. Asylum levels and trends in industrialized countries in 2010 Statistical overview of asylum applications lodged in Europe and selected non-European countries. Geneva: UNHCR; 2011.

[4] Halilovich H. Bosnian Austrians: Accidental migrants in trans-local and cyber spaces. Journal of Refugee Studies. 2013;26(4):524–40. 10.1093/jrs/fet002

[5] Fassmann H, Stacher I, editors. Österreichischer Migrations- und Integrationsbericht. Demographische Entwicklungen–sozioökonomische Strukturen–rechtliche Rahmenbedingungen [Austrian migration and integration report. Demographic developments—socioeconomic situation—legal framework]. Klagenfurt/Celovec: Drava; 2003.

[6] Franz B. Uprooted and unwanted: Bosnian refugees in Austria and the United States College Station: Texas: A&M University Press; 2005.

[7] Rosenberger S, König A. Welcoming the unwelcome: The politics of minimum reception standards for asylum seekers in Austria. Journal of Refugee Studies. 2012;25(4):537–54. 10.1093/jrs/fer051

[8] BAMF. Aktuelle Zahlen zu Asyl. Ausgabe: März 2016 [Recent numbers on asylum: March 2016]. Nürnberg: Bundesamt für Migration und Flüchtlinge [Federal Office for Migration and Refugees]; 2016.

[9] BMI. Asylstatistik 2015 [Asylum statistics 2015]. Vienna: Austrian Federal Ministry of the Interior; 2016.

[10] Eurostat: Your key to European statistics [Internet]. 2016. Available from: http://ec.europa.eu/eurostat/web/asylum-and-managed-migration/data/database.

[11] BAMF. Asylgeschäftsstatistik für den Monat Dezember 2015 [Asylum statistics for December 2015]. Nürnberg: Bundesamt für Migration und Flüchtlinge [Federal Office for Migration and Refugees]; 2016.

[12] EJPD. Asylstatistik 3. Quartal 2015 [Asylum statistics 3rd quarter 2015]. Bern-Wabern: Eidgenössisches Justiz- und Polizeidepartement EJPD [Federal Department of Justice and Police]; 2015.

[13] January—December 2015: Asylum applications lodged in Norway by Citizenship, Sex and Age [Internet]. UDI (Norwegian Directorate of Immigration). 2016. Available from: https://www.udi.no/en/statistics-and-analysis/statistics/asylum-applications-lodged-in-norway-by-citizenship-sex-and-age/.

[14] Migrationsverket. Asylum applications. Stockholm: Migrationsverket [Swedish Migration Agency]; 2016.

[15] Ruiz I, Siegel M, Vargas-Silva C. Forced up or down? The impact of forced migration on social status. Journal of Refugee Studies. 2015;28(2):183–201. 10.1093/jrs/feu035

[16] Ghattas H, Sassine AJ, Seyfert K, Nord M, Sahyoun NR. Prevalence and correlates of food insecurity among Palestinian refugees in Lebanon: Data from a household survey. PLoS ONE. 2015;10(6):e0130724 10.1371/journal.pone.0130724 26098108 PMC4476802

[17] AFAD. Syrian refugees in Turkey, 2013 Field survey results. Ankara: Turkish Disaster and Emergency Management Presidency; 2013.

[18] Brun C. Women in the local/global fields of war and displacement. Gender, Development and Technology. 2005;9(1):57–80.

[19] Robjant K, Hassan R, Katona C. Mental health implications of detaining asylum seekers: systematic review. The British Journal of Psychiatry. 2009;194(4):306–12. 10.1192/bjp.bp.108.053223 19336779

[20] Keller AS, Rosenfeld B, Trinh-Shevrin C, Meserve C, Sachs E, Leviss JA, et al Mental health of detained asylum seekers. The Lancet. 2003;362(9397):1721–3. 10.1016/S0140-6736(03)14846-5 14643122

[21] Thomas SL, Thomas SD. Displacement and health. British Medical Bulletin. 2004;69(115–127). 10.1093/bmb/ldh009 15226201

[22] Turner SW, Bowie C, Dunn G, Shapo L, Yule W. Mental health of Kosovan Albanian refugees in the UK. The British Journal of Psychiatry. 2003;182(5):444–8. 10.1192/bjp.182.5.444 12724249

[23] Aiyar S, Barkbu B, Batini N, Berger H, Detragiache E, Dizioli A, et al The refugee surge in Europe: Economic challenges. Washington DC: International Monetary Fund; 2016.

[24] Piętka-Nykaza E. ‘I want to do anything which is decent and relates to my profession’: Refugee doctors’ and teachers’ strategies of re-entering their professions in the UK. Journal of Refugee Studies. 2015;28(4):523–43. 10.1093/jrs/fev008

[25] UNHCR. A new beginning: Refugee integration in Europe Geneva: The UN Refugee Agency; 2013.

[26] Doyle L, O'Toole G. A lot to learn: Refugees, asylum seekers and post-16 learning London: British Research Council, 2013.

[27] OECD. Making integration work: Refugees and others in need of protection Paris: OECD Publishing, 2016.

[28] Derluyn I, Broekaert E. Unaccompanied refugee children and adolescents: The glaring contrast between a legal and a psychological perspective. International Journal of Law and Psychiatry. 2008;31(4):319–30. 10.1016/j.ijlp.2008.06.006. 10.1016/j.ijlp.2008.06.006 18644626

[29] Huemer J, Karnik N, Steiner H. Unaccompanied refugee children. The Lancet. 2009;373(9664):612–4. 10.1016/S0140-6736(09)60380-9. 19231617

[30] Bloch A. Methodological challenges for national and multi-sited comparative survey research. Journal of Refugee Studies. 2007;20(2):230–47. 10.1093/jrs/fem002

[31] Singh G, Clark BD. Creating a frame: A spatial approach to random sampling of immigrant households in inner city Johannesburg. Journal of Refugee Studies. 2013;26(1):126–44. 10.1093/jrs/fes031

[32] Faugier J, Sargeant M. Sampling hard to reach populations. Journal of Advanced Nursing. 1997;26(4):790–7. 10.1046/j.1365-2648.1997.00371.x 9354993

[33] Bloch A. The migration and settlement of refugees in Britain. Basingstoke: Palgrave; 2002.

[34] Clark G. Refugees and the Greenwich labour market. London: Local Economy Policy Unit, South Bank Polytechnic; 1992.

[35] UNHCR. Syrian refugee arrivals in Greece, April—September 2015, preliminary questionnaire findings. Geneva: UNHCR, 2015.

[36] Polzer Ngwato T. Collecting data on migrants through service provider NGOs: Towards data use and advocacy. Journal of Refugee Studies. 2013;26(1):144–54. 10.1093/jrs/fes034

[37] MacDonald A. Review of selected surveys of refugee populations, 2000–2014. Paper commissioned by the UNHCR. International Conference on Refugee Statistics; 7–9 October 2015, Antalya, Turkey 2015.

[38] Bock-Schappelwein J, Huber P. Zur Arbeitsmarktintegration von Asylsuchenden in Österreich [Regarding labour market integration of asylum seekers in Austria]. WIFO Monatsberichte 3/2016, Vienna: WIFO; 2016.

[39] Ceritoglu E, Yunculer HBG, Torun H, Tumen S. The impact of Syrian refugees on natives' labor market outcomes in Turkey: Evidence from a quasi-experimental design. Bonn: Discussion Paper No. 9348, IZA; 2015.

[40] Worbs S, Bund E. Qualifikationsstruktur, Arbeitsmarktbeteiligung und Zukunftsorientierungen. Ausgabe 1/2016 der Kurzanalysen des Forschungszentrums Migration, integration und Asyl des Bundesamts für Migration und Flüchtlinge [Qualification structure, labour market participation and orientation towards the future. Issue 1/2016 of short analyses of the research center on migration, integration and asylum of the Federal Office for Migration and Refugees]. Nürnberg: Bundesamt für Migration und Flüchtlinge [Federal Office for Migration and Refugees]; 2016.

[41] Berger J, Biffl G, Graf N, Schuh U, Strohner L. Ökonomische Analyse der Zuwanderung von Flüchtlingern nach Österreich [Economic analysis of influx of refugees to Austria]. Schriftenreihe Migration und Globalisierung, Krems: Donau-Universität Krems, Departement für Migration und Globalisierung; 2016.

[42] Brekke J-P, Aarset MF. Why Norway? Understanding asylum destinations Oslo: Institute for Social Research; 2009.

[43] Strand A, Akbari A, Wimpelmann Chaudhary T, Berg Harpviken K, Sarwari A, Suhrke A. Return in dignity, return to what? Review of the voluntary return programme to Afghanistan Bergen: Christian Michelsen Institute; 2008.

[44] Black R, Koser K, Munk K, Atfield G, D’Onofrio L, Tiemoko R. Understanding voluntary return London: Home Office; 2004.

[45] Studie zur Lebenssituation von Geflüchteten in Deutschland [Study on living conditions of refugees in Germany] [Internet]. Berlin: DIW (Deutsches Institut für Wirtschaftsforschung) [German Institute for Economic Research]; 2016. Available from: https://diw.de/de/diw_01.c.523741.de/themen_nachrichten/studie_zur_lebenssituation_von_gefluechteten_in_deutschland.html

[46] JES. Viele Unterschiede. Göttinger Max-Planck-Institut erforscht Vielfalt und Verschiedenheit von Flüchtlingen [Many differences. Max-Planck-Institute in Göttingen explores diversity of refugees]. Göttinger Tageblatt. 2016 28 January 2016.

[47] Lutz W, Butz WP, KC S, editors. World population and human capital in the twenty-first century. Oxford: Oxford University Press; 2014.

[48] Jacobsen K, Landau LB. The dual imperative in refugee research: Some methodological and ethical considerations in social science research on forced migration. Disasters. 2003;27(3):185–206. 10.1111/1467-7717.00228 14524045

[49] Bloch A. Survey research with refugees. Policy Studies. 2004;25(2):139–51. 10.1080/0144287042000262215

[50] Lee RM. Doing research on sensitive topics. London: Sage; 1993.

[51] Kalton G, Anderson DW. Sampling rare populations. Journal of the Royal Statistical Society Series A (General). 1986;149(1):65–82. 10.2307/2981886

[52] Vigneswaran D, Quirk J. Quantitative methodological dilemmas in urban refugee research: A case study of Johannesburg. Journal of Refugee Studies. 2013;26(1):110–6. 10.1093/jrs/fes035

[53] McMichael C, Nunn C, Gifford SM, Correa-Velez I. Studying refugee settlement through longitudinal research: Methodological and ethical insights from the Good Starts Study. Journal of Refugee Studies. 2015;28(2):238–57. 10.1093/jrs/feu017

[54] Börsch-Supan A, Jürges H, editors. The Survey of Health, Ageing and Retirement in Europe–Methodology Mannheim: Mannheim Research Institute for the Economics of Aging; 2005.

[55] Vikat A, Spéder Z, Beets G, Billari F, Bühler C, Desesquelles A, et al Generations and Gender Survey (GGS): Towards a better understanding of relationships and processes in the life course. Demographic Research. 2007;17(14):389–440.

[56] World Value Survey. World Value Survey 2016 [27 January 2016]. Available from: http://www.worldvaluessurvey.org/wvs.jsp.

[57] Temple B, Moran R, editors. Doing research with refugees: Issues and guidelines Bristol: The Policy Press; 2006.

[58] Hynes P. The issue of "trust" or "mistrust" in research with refugees: Choices, caveats and considerations for researchers Geneva: UNHCR; 2003.

[59] Daniel EV, Knudsen JC, editors. Mistrusting refugees Berkeley, Los Angeles and London: University of California Press; 1995.

[60] Statistical Office of the Republic of Syria. Available from: http://www.cbssyr.sy/index-EN.htm.

[61] Niessen J. Diversity and cohesion: New challenges for the integration of immigrants and minorities. Strasbourg: Council of Europe; 2001.

[62] Grogger J, Hanson GH. Income maximization and the selection and sorting of international migrants. Journal of Development Economics. 2011;95(42–57).

[63] Asylberechtigte auf Jobsuche. Kompetenzcheck-Ergebnisse und Integrationsmaßnahmen im Jahr 2016 [Persons granted asylum looking for a job. Competence check results and integration measures in the year 2016] [Internet]. Vienna; 2016. Available from: http://www.ams.at/_docs/Pressekonferenz-Asylberechtigte-auf-Jobsuche-12-01-2016.pdf

[64] Kirilova S, Biffl G, Pfeffer T, Skrivanek I, Egger-Subotitsch A, Kerler M, et al Anerkennung von Qualifikationen. Fakten, Erfahrungen, Perspektiven [Accreditation of qualifications Facts, experiences, perspectives]. ÖIF-Forschungsbericht [Research report], Vienna: Austrian Integration Fund (ÖIF); 2016.

[65] Central Statistics Organisation. National risk and vulnerability assessment 2011–2012. Afghanistan Living Conditions Survey Kabul: CSO; 2014.

[66] MICS. Iraq multiple indicator cluster survey 2011, Final Report. Baghdad, Iraq: The Central Statistics Organization and the Kurdistan Regional Statistics Office, 2012.

[67] Shisheva M, Christie G, Mulvey G. Improving the lives of refugees in Scotland after the referendum: An appraisal of the options. Glasgow: Scottish Refugee Council, 2013.

[68] Ambugo E, Yahirun J. Remittances and risk of major depressive episode and sadness among new legal immigrants to the United States. Demographic Research. 2016;34(8):243–58.

[69] Richardson S, Stack S, Lester L, Healy J, Ilsley D, Horrocks J. The changing labour force experience of new migrants. Inter-wave comparisons for cohort 1 and 2 of the LSIA Report to the Department of Immigration and Multicultural Affairs. Adelaide: National Institute for Labour Studies, Flinders University; 2004.

[70] Aydemir A. Immigrant selection and short-term labor market outcomes by visa category. Journal of Population Economics. 2011;24(2):451–75. 10.1007/s00148-009-0285-0

[71] Bevelander P. The employment integration of resettled refugees, asylum claimants, and family reunion migrants in Sweden. Refugee Survey Quarterly. 2011;30(1):22–43. 10.1093/rsq/hdq041

[72] Cortes KE. Are refugees different from economic immigrants? Some empirical evidence on the heterogeneity of immigrant groups in the United States. Review of Economics and Statistics. 2004;86(2):465–80. 10.1162/003465304323031058

[73] Refugee Council of Australia. Economic, civic and social contributions of refugees and humanitarian entrants—A literature review. Canberra: Refugee Council of Australia; 2010.

[74] Hartog J, Zorlu A. How important is homeland education for refugees’ economic position in The Netherlands? Journal of Population Economics. 2009;22(1):219–46. 10.1007/s00148-007-0142-y

[75] Statistics Austria. Mikrozensus-Arbeitskräfteerhebung 2014: Unselbständig Erwerbstätige (ILO) nach ÖNACE und Geschlecht, 2014 [Microcensus-Labour Force Survey 2014: Employees (ILO) by ÖNACE and gender, 2014] 25 January 2016. Available from: http://statistik.at/web_de/statistiken/menschen_und_gesellschaft/soziales/gender-statistik/erwerbstaetigkeit/043909.html.

[76] BMEIA. 50 Punkte–Plan zur Integration von Asylberechtigten und subsidiär Schutzberechtigten in Österreich [50 point plan for the integratin of persons granted asylum and subsidiary protection in Austria]. Vienna: Austrian Federal Ministry for Europe, Integration and Foreign Affairs; 2015.

[77] Domnich A, Panatoo D, Gasparini R, Amicizia D. The “healthy immigrant” effect: Does it exist in Europe today? Italian Journal of Public Health. 2012;9(3):e7532–1-e-7.

[78] Marmot MG, Adelstein AM, Bulusu L. Lessons from the study of immigrant mortality. The Lancet. 1984;323(8392):1455–7. 10.1016/s0140-6736(84)91943-3 6145889

[79] Kohls M. Morbidität und Mortalität von Migranten in Deutschland [Morbidity and Mortality of migrants in Germany]. Nürnberg: Bundesamt für Migration und Flüchtlinge [Federal Office for Migration and Refugees]; 2011.

[80] Benyamini Y, Blumstein T, Lusky A, Modan B. Gender differences in self-rated health-mortality association: Is it poor self-rated health that predicts mortality or excellent self-rated health that predicts survival? The Gerontologist. 2003;43(396–405). 10.1093/geront/43.3.396 12810904

[81] BMG. Österreichische Gesundheitsbefragung 2014. Hauptergebnisse des Austrian Health Interview Survey (ATHIS) und methodische Dokumentation [Austrian Health Survey 2014. Main results of the Austrian Health Interview Survey (ATHIS) and methodological documentation]. Vienna: Austrian Federal Ministry of Health; 2015.

[82] Fetzer JS, Soper C. Muslims and the state in Britain, France, and Germany Cambridge: Cambridge University Press; 2005.

[83] Bawer B. While Europe slept: How radical Islam is destroying the West from within. New York: Broadway Books; 2007.

[84] Norris P, Inglehart R. Sacred and secular: Religion and politics worldwide. Cambridge: Cambridge University Press; 2011.

[85] Inglehart R, Norris P. The true clash of civilizations. Foreign Policy. 2003;135(62). 10.2307/3183594

[86] Carrera S, Blockmans S, Gros D, Guild E. The EU’s response to the refugee crisis: Taking stock and setting policy priorities. Brussels: Centre for European Policy Studies, 2015.

[87] Culik J. Anti-immigrant walls and racist tweets: the refugee crisis in central Europe. The Conversation. 24 June 2015.

[88] Croucher SM, Cronn-Mills D. Religious misperceptions: The case of Muslims and Christians in France and Britain. New York: Hampton Press; 2011.

[89] Dearing H. Gender equality in the division of work—how to assess European leave policies regarding their compliance with an ideal leave model. Journal of European Social Policy. Forthcoming.

[90] OECD. OECD economic surveys: Austria: OECD Publishing; 2015.

[91] Röder A, Mühlau P. Are they acculturating? Europe’s immigrants and gender egalitarianism. Social Forces. 2014;92(3):899–928. 10.1093/sf/sot126

[92] Norris P, Inglehart RF. Muslim Integration into Western Cultures: Between Origins and Destinations. Political Studies. 2012;60(2):228–51. 10.1111/j.1467-9248.2012.00951.x

[93] Gholami R. Secularism and identity: Non-Islamiosity in the Iranian Diaspora. Surrey: Ashgate Publishing Limited; 2015.

[94] Trezebiatowska M, Bruce S. Why are women more religious than men? Oxford: Oxford University Press; 2012.

[95] Johnson DC. Formal education vs. religious belief: Soliciting new evidence with multinomial logit modeling. Journal for the Scientific Study of Religion. 1997:231–46.

[96] Burda M, Hamermesh DS, Weil P. Total work and gender: Facts and possible explanations. Journal of Population Economics. 2012;26(1):239–61. 10.1007/s00148-012-0408-x

[97] Hall A, Zoega G. Values and labor force participation in the Nordic countries. Economics: The Open-Access, Open-Assessment E-Journal. 2014;8(2014–41):1–43.

[98] Blau FD, Kahn LM, Yung-Hsu Liu A, Papps KL. The transmission of women’s fertility, human capital, and work orientation across immigrant generations. Journal of Population Economics. 2013;26(2):405–35.

[99] Antecol H. Why is there interethnic variation in the gender wage gap?: The role of cultural factors. The Journal of Human Resources. 2001;36(1):119–43. 10.2307/3069672

[100] Dijkstra W. How interviewer variance can bias the results of research on interviewer effects. Qual Quant. 1983;17(3):179–87.

[101] Kane EW, Macaulay LJ. Interviewer gender and gender attitudes. Public Opinion Quarterly. 1993;57(1):1–28. 10.1086/269352

[102] Read JG. The sources of gender role attitudes among Christian and Muslim Arab-American women. Sociology of Religion. 2003;64(2):207–22. 10.2307/3712371

[103] Stefanovic D, Loizides N, Parsons S. Home is where the heart is? Forced migration and voluntary return in Turkey’s Kurdish regions. Journal of Refugee Studies. 2015;28(2):276–96. 10.1093/jrs/feu029

[104] Billiet J, Philippens M, Fitzgerald R, Stoop I. Estimation of nonresponse bias in the European Social Survey: Using information from reluctant respondents. Journal of Official Statistics. 2007;23(2):135–62.

[105] Festy P, Prioux F. An evaluation of the Fertility and Family Surveys project. New York/Geneva: United Nations; 2002.

[106] Kreyenfeld M, Zeman K, Burkimsher M, Jaschinski I. Fertility data for German-speaking countries: What is the potential? Where are the pitfalls? Comparative Population Studies/ Zeitschrift für Bevölkerungswissenschaft. 2011;36(2–3):349–80. 10.4232/10.CPoS-2011-06en

[107] Miller RB, Wright DW. Detecting and correcting attrition bias in longitudinal family research. Journal of Marriage and Family. 1995;57(4):921–9.

[108] Haisken-DeNew JP, Frick JR, editors. DTC Desktop companion to the German Socio-Economic Panel (SOEP) Berlin: DIW; 2005.

