# Supplementary figures and images for: Human Capital, Values, and Attitudes of Persons Seeking Refuge in Austria in 2015
Source: PLoS One. 2016 Sep 23;11(9):e0163481. doi: 10.1371/journal.pone.0163481 (PMC5035031; doi:10.1371/journal.pone.0163481)

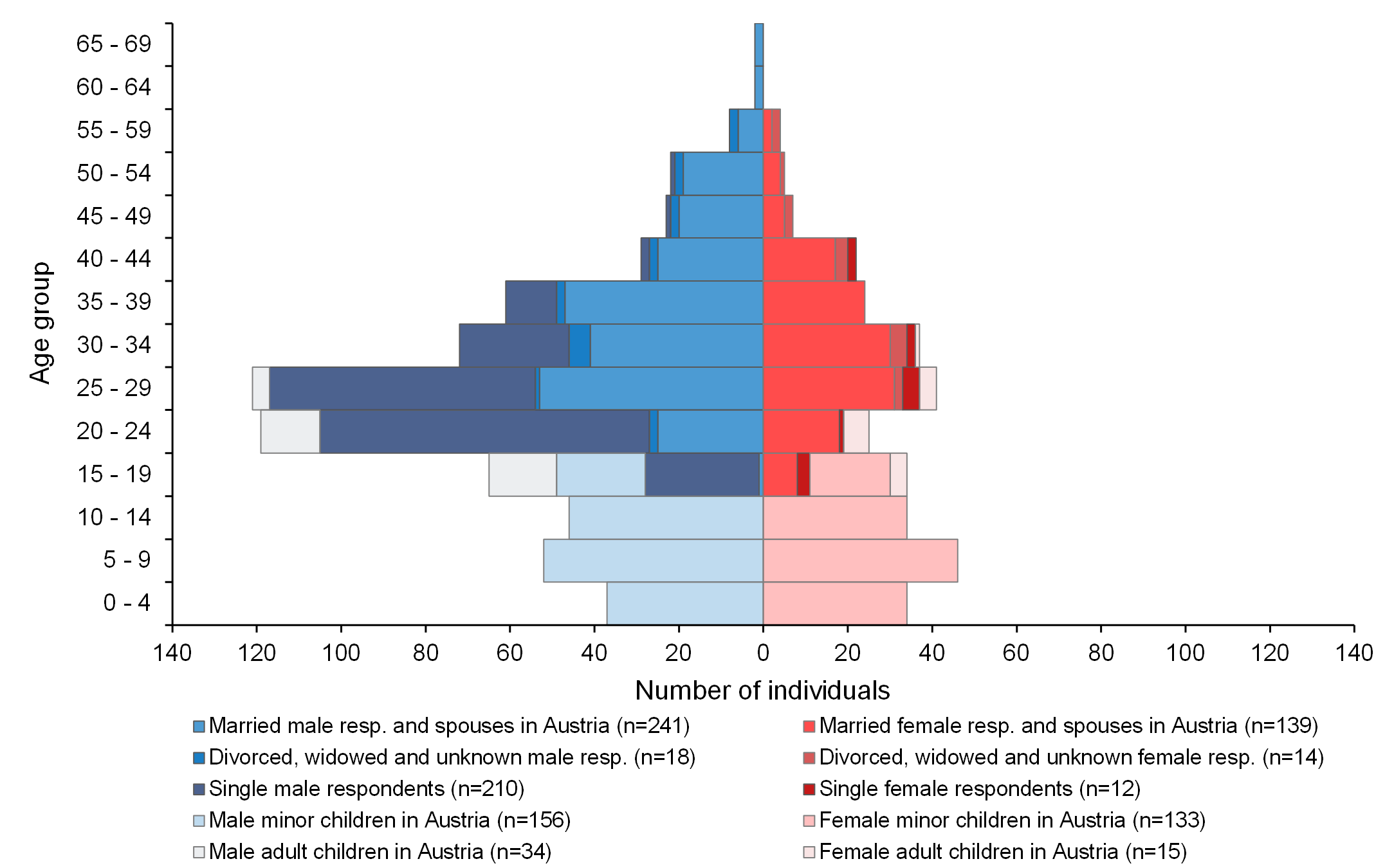

Supplement: S1 Fig — Source: DiPAS, n = 972 individuals captured in the survey, living in Austria. (TIF) [file pone.0163481.s002.tif]
