# Supplementary material for: Human Capital, Values, and Attitudes of Persons Seeking Refuge in Austria in 2015
Source: PLoS One. 2016 Sep 23;11(9):e0163481. doi: 10.1371/journal.pone.0163481 (PMC5035031; doi:10.1371/journal.pone.0163481)
Supplement: S1 File — (DOCX) [file pone.0163481.s003.docx]

**S1 Supporting material: Historical and comparative perspective on the flows of displaced persons to Austria**

According to asylum statistics by the Austrian Federal Ministry of the Interior, 88,098 individuals applied for asylum in Austria in 2015 (BMI 2016a). In comparison, during the years 2014 and 2013, a total of 28,064 and 17,503 asylum applications were recorded, respectively. The last peak in this century took place during the years 2001-2003, when approximately 30,000 to 40,000 applications were received annually (Figure S1a).

#### Figure S1a: Asylum applications in Austria since 1999

Source: BMI (2013, 2016a).

Although in 2015 the majority of arriving persons regarded Austria as a transit country on their way to Germany or Scandinavia, asylum applications still increased noticeably in the second half of the year from a monthly figure of 3,000-4,000 (January-April), to 6,000-8,000 (May-June) and to 9,000-12,000 (July-December) (Figure S1b). Monthly statistics show that the autumn of 2015 was the season with the highest number of asylum applications.

In 2001, a total of 30,127 persons applied for asylum, the largest group being Afghans. In 2002, the largest groups were persons from Afghanistan, Serbia and Montenegro, Iraq and Turkey. In 2003, asylum seekers from the Russian Federation comprised by far the largest group, followed by persons from Turkey and India (BMI 2004; Statistics Austria 2015a).

#### Figure S1b: Asylum applications in Austria in 2014, 2015 and 2016 by nationality and month of application

Source: BMI (2015, 2016a, b).

The Austrian Federal Ministry of the Interior publishes monthly asylum statistics, which include asylum seekers’ nationalities and hence show the development of this demographic over time (Figure S1b). As the field phase of the Displaced Persons in Austria Survey (DiPAS) was in November, we briefly focus on September and October 2015. During these two months, one-third of asylum seekers were Syrian, 21% were Iraqi and approximately one-quarter (27%) were Afghan, while the nationality of the remaining 18% was given as “other”. In other words, 8 out of 10 asylum seekers who arrived in September and October 2015 originate from Syria, Iraq or Afghanistan. The composition of asylum seekers by citizenship varies over time. In 2014, 28% of all asylum seekers were Syrian, 4% Iraqi, and 18% Afghan, while half (50%) came from other countries. In 2014, the composition of asylum seekers’ nationalities was substantially different. Moreover, the citizenship of asylum seekers varies greatly between countries. As an example, Figure S1c visualizes the citizenship of asylum seekers in the three neighbouring German-speaking countries Germany, Austria, and Switzerland as well as Norway and Sweden.

#### Figure S1c: Comparison of the citizenship of asylum seekers arriving in Austria, Germany, Switzerland, Sweden and Norway in 2015

Sources: BMI (2016a), BAMF (2016), EJPD (2016), UDI (2016), Migrationsverket (2016)

Remark: For Germany and Switzerland available data include not all nationalities and are restricted to the ten most frequent nationalities.

**References**

BAMF. 2016. *Asylgeschäftsstatistik Für Den Monat Dezember 2015 [Asylum Statistics for December 2015].* Nürnberg: Bundesamt für Migration und Flüchtlinge [Federal Office for Migration and Refugees].

BMI. 2013. *Asylanträge Seit 1999 [Asylum Applications since 1999].* Vienna: Austrian Federal Ministry of the Interior.

BMI. 2015. *Asylstatistik Dezember 2014 [Asylum Statistics December 2014].* Vienna: Austrian Federal Ministry of the Interior.

BMI. 2016a. *Asylstatistik 2015 [Asylum Statistics 2015].* Vienna: Austrian Federal Ministry of the Interior.

BMI. 2016b. *Vorläufige Asylstatistik April 2016 [Preliminary Asylum Statistics April 2016].* Vienna: Austrian Federal Ministry of the Interior.

EJPD. 2016. *Asylstatistik 2015 [Asylum Statistics 2015].* Bern-Wabern: Eidgenössisches Justiz- und Polizeidepartement EJPD [Federal Department of Justice and Police] (<https://www.sem.admin.ch/dam/data/sem/publiservice/statistik/asylstatistik/2015/stat-jahr-2015-kommentar-d.pdf)>.

Migrationsverket. 2016. *Asylum Applications.* Stockholm: Migrationsverket [Swedish Migration Agency] (<http://www.migrationsverket.se/download/18.7c00d8e6143101d166d1aab/1451894593595/Inkomna+ans%C3%B6kningar+om+asyl+2015+-+Applications+for+asylum+received+2015.pdf)>.

UDI. 2016. "Asylum Applications Lodged in Norway by Citizenship and Month (2015)." Oslo: UDI (Norwegian Directorate of Immigration).
