# Supplementary material for: Human Capital, Values, and Attitudes of Persons Seeking Refuge in Austria in 2015
Source: PLoS One. 2016 Sep 23;11(9):e0163481. doi: 10.1371/journal.pone.0163481 (PMC5035031; doi:10.1371/journal.pone.0163481)
Supplement: S2 File — (PDF) [file pone.0163481.s004.pdf]

Refugees in Austria: Human Capital and Potential for Integration

A survey on asylum seekers and refugees

Wittgenstein Centre for Demography and Global Human Capital,
Vienna University of Economics and Business
Welthandelsplatz 2, 1020 Wien

November 2015

Q0.1 Interviewer: Please choose the facility / location at which you are conducting the interview (site)

Q0.1a Interviewer: Please enter the facility / location at which you are conducting the interview (site) if not in the previous list

Q0.2 Interviewer: Please select the name of the main interviewer (the one asking the questions)

Q0.3 Interviewer: Please select the name of the assistant interviewer (entering data) if applicable

Questionnaire

This is a scientific survey whose purpose is to gain demographic information about the refugee population in Austria. The project is conducted by independent academic researchers from the Wittgenstein Centre for Demography and Global Human Capital and students from the Vienna University of Economics and Business.
Please be assured that all your responses are confidential and anonymous, and will not affect your application for asylum in Austria. We will not forward your data to third parties. All results will be exclusively used for the purpose of the current survey.

Q1 Interviewer: Please enter the sex of the interviewee

2 Female
1 Male

Q2 How old are you?

(code age in years)

Q3 Are you seeking asylum or have you already been granted asylum in Austria?

1 Asylum process in Austria (white card)
2 Asylum seeking (green card)
3 Subsidiary protection (grey card)
4 Refugee (holding a convention passport)
5 Temporary suspension of deportation (yellow card)

6 No status yet (no card)
99 No answer / refusal

Q4 In which country were you born?

1 Syria
2 Iraq
3 Turkey
4 Libya
5 Jordan
6 Lebanon
7 Iran
8 Afghanistan
99 No answer / refusal
77 Other (please specify->code as 4b)

Q5 Which country was your last place of residence?
(Note: Not the country of refugee camp)

1 Syria
2 Iraq
3 Turkey
4 Libya
5 Jordan
6 Lebanon
8 Afghanistan
11 Pakistan
12 Kosovo
13 Somalia
7 Iran
99 No answer / refusal
77 Other (please specify->code as 4b)

Q6 What is your citizenship?

1 Syria -> go to 7a
2 Iraq -> go to 7b
15 Palestinian in Syria -> go to 7a
16 Palestinian in Iraq -> go to 7b
3 Turkey -> go to 8
8 Afghanistan -> go to 7c
11 Pakistan -> go to 8
12 Kosovo -> go to 8
7 Iran -> go to 8
14 Stateless -> go to 8
99 No answer / refusal -> go to 8
77 Other (please specify -> code as 6b) -> go to 8

Q7a Where in Syria have you lived most of your life?(Governorates)

1 Damascus and suburbs
2 Aleppo
3 Qamishli
4 Deir ez-zor
5 Hama

6 Al-hasakah
7 Homs
8 Idlip
9 Latakia
10 Tartus
11 Asuwyda
12 Alraqqah
13 Daraa
14 Al Quneitra
99 No answer / refusal

Q7b Where in Iraq have you lived most of your life? (Governorates)

1 Anbar
2 Babylon
3 Baghdad
4 Basra
5 Dhi Qar
6 Diyala
7 Dohuk
8 Erbil
9 Halabja
10 Karbala
11 Kirkuk
12 Maysan
13 Muthanna
14 Najaf
15 Nineveh
16 Saladin
17 Sulaymaniyah
18 Wasit
99 No answer / refusal

Q7c Where in Afghanistan have you lived most of your life? (Provinces)

1 Badakhshan
2 Badghis
3 Baghlan
4 Balkh
5 Bamyan
6 Daykundi
7 Farah
8 Faryab
9 Ghazni
10 Ghor
11 Helmand
12 Herat
13 Jowzjan
14 Kabul
15 Kandahar
16 Kapisa
17 Khost
18 Kunar

19 Kunduz
20 Laghman
21 Logar
22 Maidan Wardak
23 Nangarhar
24 Nimruz
25 Nuristan
26 Paktia
27 Paktika
28 Panjshir
29 Parwan
30 Samangan
31 Sar-e Pol
32 Takhar
33 Urozgan
34 Zabul
99 No answer / refusal

Q8 **What type of residence did you live in before you came here?**

1 Your own home
2 Your family's home
3 A rental home
99 No answer / refusal
77 Other

Q9 **What is your ethnicity?**

1 Arab
2 Kurdish
3 Turkmen
4 Afghan
99 No answer / refusal
77 Other (please specify -> code as 9b)

Q10 **What is your religion?**

1 Islam
2 Christian
3 None (e.g. Atheist)
99 No answer / refusal
77 Other

Q11 **What is your highest level of educational attainment?**

1 No formal education (never been to school)
2 Some primary (elementary)
3 Completed primary (6 years)
4 Completed lower secondary (In Syria called "intermediate") (9 years)
5 Completed upper secondary (12 years)
6 Bachelor or college
7 Master and more
99 No answer / refusal

Q12 If 11 is (5), (6) or (7): **In which field did you study?**

1 Science
2 Humanities (History, Law,…)
3 Technical (including e.g. industrial, agriculture, craft)
4 Islamic law school/studies
5 Commerce and Economics
99 No answer / refusal
77 Other (what exactly: code as 12c)

Q13 How many years of education have you completed?
(in years)

Q14 Do you speak other languages than your mother tongue?
1 Yes -> go to 15
2 No -> go to 16
99 No answer / refusal -> go to 16

Q15 Which other language(s) do you speak?
Q15_en 1 English
Q15_de 1 German
Q15_fr 1 French
Q15_ku 1 Kurdish
Q15_tr 1 Turkish
Q15_99 1 No answer / refusal
Q15_77 Others (please specify: code as 15b, 15c,…)

Q16 Did you ever actively participate in the labor market?
1 Yes -> go to 17
2 No -> go to 22
99 No answer / refusal -> go to 22

Q17 What was your main occupation before the beginning of the war or before your situation forced you to flee from your country
(Please enter ISCO 2 digit code)

Q17a Please enter the name/description of the occupation

Q18 In which economic branch have you been active or employed?
(Please enter NACE code)

Q19 Which best describes the work status of the job specified?
1 Employed
2 Self-employed
3 Working with/ for family member in a family business or a farm
77 Other
99 No answer / refusal

Q20a1 Did you work in other occupations for more than six months?
1 Yes -> go to 20a2, 20b2, 20c2
2 No -> go to 21
99 No answer / refusal -> go to 21

Q20a2 **Please enter the ISCO code of this other occupation**
(Please enter ISCO 2 digit code)

Q20a3 **Please enter the name/description of this other occupation**

Q21 **How many hours did you usually work each week?**
1 5 to 9 hours (1 day per week) -> go to 25
2 10 to 19 hours (2 days per week) -> go to 25
3 20 to 34 hours (3-4 days per week) -> go to 25
4 35 or more hours (more than 4 days per week) -> go to 25
5 Seasonal work -> go to 25
99 No answer / refusal -> go to 25

Q22 **Have you ever received job training?**
1 Yes -> go to 23
2 No -> go to 25
99 No answer / refusal -> go to 25

Q23 **For which profession/job did you receive the most extensive training?**
(Please enter ISCO 2 digit code)

Q23a **Please enter the name/description of the profession/job that you were trained in**

Q24 **Did you complete this job training?**
1 Yes
2 No
99 No answer / refusal

Q25 **What do you plan to do in the future after you received a status (asylum or subsidiary protection)?**
1 Search for a job -> go to 26
2 To continue school/studying -> go to 28
88 Do not know -> go to 28
99 No answer / refusal -> go to 28

Q26 **Would you be willing to work in a different position or sector than in those you were active so far if you get the training?**
1 Yes
2 No
88 Do not know
99 No answer / refusal

Q27 **How many hours do you want to work each week?**
1 Less than 20 hours
2 20 to 34 hours
3 35 or more hours
99 No answer / refusal

Measure grip strength

Now I would like to assess the strength of your hand in a gripping exercise. I will ask you to squeeze this handle as hard as you can, just for a couple of seconds, and then let go. I will take two alternate measurements from your right and your left hand.

Q28a **First measurement, left hand.**
Interviewer: Enter the results to the nearest integer value.

Q28b **Second measurement, left hand.**
terviewer: Enter the results to the nearest integer value

Q28c **First measurement, right hand.**
Interviewer: Enter the results to the nearest integer value.

Q28d **Second measurement, right hand.**
Interviewer: Enter the results to the nearest integer value.

Q29 **How tall are you?**
(code height in cm)

Q30 **What is your general assessment of your health?**
1 Very good
2 Good
3 Neither good nor bad
4 Bad
5 Very bad
99 No answer / refusal

Q31 **Do you have any long-term (chronic) illness or health problems?**
1 Yes
2 No
99 No answer / refusal

Q32 **Has any health problem restricted the performance of your everyday activities over a long time (at least in the last 6 months)?**
1 Yes, very much
2 Yes, to some extent
3 No, not at all
99 No answer / refusal

Q33 **When did you arrive in Austria?**
Date

Q34 **How long did it take you to arrive in Austria?**
Q34a Days
Q34b Weeks
Q34c Months
Q34d Years

Q35 **How did you come to Austria?**
1 Through Turkey

2 Through Libya
99 No answer / refusal
77 Other -> Please specify (code as 34b)

Q36 **How much did your journey to Austria cost (per person)?**

1 < 2000 $
2 >=2000 $ <3000 $
3 >=3000 $ <4000 $
4 > 4000 $
99 No answer / refusal
77 Much more: Please specify (code as 36b)

Q37 **Would you consider to return to your home country after the situation has stabilized?**

1 Yes -> go to 38
2 No -> go to 37a
88 Do not know -> go to 37a
99 No answer / refusal -> go to 38

Q37a **Could you please specify the reasons? (max 3 reasons)**

Q37a_1 1 I don’t think that the general political situation will stabilize within the next years
Q37a_2 1 I don’t think that the personal safety of my family and me would be guaranteed, even if the general situation stabilizes
Q37a_3 1 I don’t think that the economic situation will stabilize within the next years
Q37a_4 1 I don’t think that I will be able to make a living in my home country, even if the situation stabilizes

Q37a_5 1 My reasons for leaving my home country were not related to instabilities in my home country
Q37a_6 1 I have nowhere/no one to return to
Q37a_7 1 I have no more emotional attachment to my home country
Q37a_8 1 Even if I wanted to, I couldn’t afford to return (only had enough money to come here)

Q37a_88 1 Do not know
Q37a_99 1 No answer / refusal
Q37a_77 Other (brief answer -> code as 37b)

Q38 **Did you lose any family member since the beginning of the war/since your situation forced you to flee?**

1 Yes -> go to 39
2 No -> go to 40
99 No answer / refusal -> go to 40

Q39 **Whom did you lose? (Multiple answers, code as 38a, 38b, …)**

Q39_F 1 Father
Q39_M 1 Mother
Q39_W 1 Wife
Q39_H 1 Husband
Q39_SD 1 Son or Daughter
Q39_BS 1 Brother or sister

Q39_77 1 Others
Q39_99 1 No answer / refusal

Q40 **What is your marital status?**

1 Married -> go to 41
2 Widowed -> go to 49
3 Divorced -> go to 49
4 Single -> go to 52
99 No answer / refusal -> go to 52

Q41 **How old is your wife/husband?**

(code in years)

Q42 **Where does your wife/husband currently live?**

20 He/she is with me
1 Syria
2 Iraq
3 Turkey
8 Afghanistan
11 Pakistan
12 Kosovo
13 Somalia
10 Austria
15 On the way to Europe/US/other region
77 Other country
88 Do not know
16 Camp -> in which country (code as 42b)

Q43 **What is your wife's/husband's highest level of educational attainment?**

1 No formal education (never been to school)
2 Some primary (elementary)
3 Completed primary (6 years)
4 Completed lower secondary (In Syria called “intermediate”) (9 years)
5 Completed upper secondary (12 years)
6 Bachelor or college
7 Master and more
88 Do not know
99 No answer / refusal

Q44 If 43 is (5), (6) or (7): **In which field did your wife/husband study?**

1 Science
2 Humanities (History, Law,…)
3 Technical (including e.g. industrial, agriculture, craft)
4 Islamic law school/studies
5 Commerce and Economics
88 Do not know
99 No answer / refusal
77 Other (what exactly: code as 44b)

Q45 **Did your wife/husband ever actively participate in the labor market?**

1 Yes -> go to 46

2 No -> go to 49
88 Do not know -> go to 49
99 No answer / refusal -> go to 49

Q46 What was your wife's/husband's main occupation before the beginning of the war or before your situation forced you to flee from your country?

(Please enter ISCO 2 digit code)

Q46a Please enter the name/description of your wife's/husband's occupation

Q47 In which economic branch was your wife/husband active or employed?

(Please enter NACE code)

Q48 Which best describes the work status of the job specified?

1 Employed
2 Self-employed
3 Working with/for family member in a family business or a farm
77 Other
88 Do not know
99 No answer / refusal

Q49 Has your husband/wife ever received job training?

1 Yes -> go to 50
2 No -> go to 52
88 Do not know -> go to 52
99 No answer / refusal -> go to 52

Q50 For which profession/job did your husband/wife receive the most extensive training?

(Please enter ISCO 2 digit code)

Q50a Please enter the name/description of the profession/job that your husband/wife was trained in

Q51 Did your husband/wife complete this job training?

1 Yes
2 No
88 Do not know
99 No answer / refusal

Q52 Do you have any children?

1 Yes -> goto 52a
2 No -> goto 59
99 No answer / refusal -> goto 59

Q52a How many children do you have?

May I now ask you some details about your first child?

Q53 First Name

Q54 How old is [name]?

Q55 Sex of [name]

1 Male
2 Female

Q56 Where does [name] currently live?

20 Here with me
1 Syria
2 Iraq
3 Turkey
8 Afghanistan
11 Pakistan
12 Kosovo
13 Somalia
10 Austria
15 On the way to Europe/US/other region
77 Other country
88 Do not know
16 Camp -> in which country (code as 56b)

Q57 What is the educational level of [name]?

1 No formal education (never been to school)
2 Some primary (elementary)
3 Completed primary (6 years)
4 Completed lower secondary (In Syria called "intermediate") (9 years)
5 Completed upper secondary (12 years)
6 Bachelor or college
7 Master and more
99 No answer / refusal

Q58 Interviewer: Describe another child?

1 Yes -> go to 53a, go to 53b, go to 53c, …
2 No more children -> go to 59
3 Interviewee does not wish to continue -> go to 59

The following statements were selected from the World Value Survey, a non-commercial, scientific investigation which seeks to help understand changes in the beliefs, values and motivations of people throughout the world. There is no "right" or "wrong" answer to these questions. Please note that we do not ask for your country's official or legal stance on the following statements nor should your response be guided by social conventions and expectations. Rather, we are interested in your personal beliefs and values. We would like to stress that your answers to these questions are completely confidential and will not affect your application for asylum in Austria in any way – our survey is conducted for purely scientific purposes and none of the data your provide us with will be forwarded to third parties.

To what extent do you agree or disagree with each of the following statements? For each question choose between:

5 Strongly agree
4 Agree
3 Neither agree/nor disagree
2 Disagree
1 Strongly disagree
99 No answer / refusal

Q59 When jobs are scarce, men should have more right to a job than women.

5 Strongly agree
4 Agree
3 Neither agree/nor disagree
2 Disagree
1 Strongly disagree
99 No answer / refusal

Q60 Men make better political leaders than women do.

5 Strongly agree
4 Agree
3 Neither agree/nor disagree
2 Disagree
1 Strongly disagree
99 No answer / refusal

Q61 Having a job is the best way for a woman to be an independent person.

5 Strongly agree
4 Agree
3 Neither agree/nor disagree
2 Disagree
1 Strongly disagree
99 No answer / refusal

Q62 Women should not work outside the family unless forced to do so (i.e. women should only work outside the family if forced to do so).

5 Strongly agree
4 Agree
3 Neither agree/nor disagree
2 Disagree
1 Strongly disagree
99 No answer / refusal

Q63 Men make better bosses than women.

5 Strongly agree
4 Agree
3 Neither agree/nor disagree
2 Disagree
1 Strongly disagree
99 No answer / refusal

Q64 Please tell me whether you think abortion can always be justified, never be justified, or something in between.
(1 = never, … , 10 = always)

1 1 Never
2 2
3 3
4 4
5 5
6 6
7 7
8 8
9 9
10 10 Always

Q65 Would you mind if your children learned about other religions in school?

1 It would be ok
2 I do not care
3 I would not want that
88 I do not know
99 No answer / refusal

Q66 Apart from the fact of belonging to a religious community or not, how religious do you consider yourself? Please answer using a scale, where 0 indicates "not at all religious" and 10 "very religious".

1 1 not at all religious
2 2
3 3
4 4
5 5
6 6
7 7
8 8
9 9
10 10 very religious

Interviewer: Please select the family status that the interviewee stated during the interview (i.e. if he/she is married, widowed or divorced)

1 **Single -> go to 72**
2 **Not single (married, widowed or divorced) -> go to 67**

The following questions are also taken from non-commercial surveys with an exclusively scientific purpose. They ask about your family's decision-making in concrete situations, such as everyday shopping, childcare or the distribution of household work.

Q67 Who usually makes the decisions or takes actions in your household when buying rather expensive household items e.g. fridge, tv

1 Mostly the husband
2 Mostly the wife
3 Husband and wife together

4 Children
5 Parents
6 Everyone
7 Another person
88 I do not know
99 No answer / refusal

In your household who does the following things?

Q68 Cares for sick family members

1 Mostly the husband.
2 Mostly the wife
3 Husband and wife together.
4 Children
5 Parents
6 Everyone
7 Another person.
88 I do not know
99 No answer / refusal

Q69 Childcare

1 Mostly the husband.
2 Mostly the wife
3 Husband and wife together.
4 Children
5 Parents
6 Everyone
7 Another person.
88 I do not know
99 No answer / refusal

Q70 Does the household chores

1 Mostly the husband.
2 Mostly the wife
3 Husband and wife together.
4 Children
5 Parents
6 Everyone
7 Another person.
88 I do not know
99 No answer / refusal

Q71 Keep the household account

1 Mostly the husband.
2 Mostly the wife
3 Husband and wife together.
4 Children
5 Parents
6 Everyone
7 Another person.
88 I do not know

99 No answer / refusal

Q72 We have now come to the end of our interview. Thank you very much for your cooperation! May we interview you again in the future - in about six months or later?

1 Yes -> Go to 73
2 No -> Go to 74

Q73 Could you please give us your E-Mail address, Facebook address, other social network account name, telephone number or your name so that we can contact you? We will not hand this information to any other person!

2 No
1 Yes -> please enter email or username and service name (e.g. facebook: username)

Interviewer: This is the end of the interview for the interviewee!
Please thank the interviewee again and fill in the following questions immediately after he/she left.
Before starting a new interview do not forget to submit this survey, start a new one and fill in the first page with the location and interviewer information.

Q74 Interviewer: Please describe the interview situation

1 Separate room only one interview at the same time
2 Separate room with multiple interviews
3 Open room with other people around
4 Living Quarters
77 Other Situation

Q74a Interviewer: Who participated in the interview (was in hearing range)

1 Only interviewee
2 Interviewee and his/her partner (husband/wife)
3 Interviewee and family (children present)
4 Interviewee and others in hearing range
77 Other Situation

Q74b Interviewer: Please enter the name of the translators present

Q74c Interviewer: Please enter the name(s) of other translators or Interviewers that participated

Q74d Interviewer: Please choose the language in which the Interview was mainly conducted

2 English
1 Arabic
3 Dari / Farsi
77 Other

Q74e Interviewer: who answered questions 59 - 71 (attitudes and practices)?

1 The interviewee alone
2 The partner of the interviewee alone
3 Both partners together

4 The whole family
5 The interviewee and bystanders
77 Other situation (please specify)

Notes researcher
