# Supplementary material for: Human Capital, Values, and Attitudes of Persons Seeking Refuge in Austria in 2015
Source: PLoS One. 2016 Sep 23;11(9):e0163481. doi: 10.1371/journal.pone.0163481 (PMC5035031; doi:10.1371/journal.pone.0163481)
Supplement: S1 Table — Source: Displaced Persons in Austria Survey (DiPAS), n = 514 interviewed persons. (DOCX) [file pone.0163481.s005.docx]

#### S1 Table. Characteristics of respondents.

|  | **All respondents** | | **Share by citizenship subgroups** | | | |
| --- | --- | --- | --- | --- | --- | --- |
|  | **Absolute** | **Relative** | **Iraq** | **Syria** | **Afghanistan** | **Other** |
| **Citizenship** |  |  |  |  |  |  |
| Iraq | 196 | 38% |  |  |  |  |
| Syria | 184 | 36% |  |  |  |  |
| Afghanistan | 83 | 16% |  |  |  |  |
| Other | 51 | 10% |  |  |  |  |
| **Gender** |  |  |  |  |  |  |
| Male | 419 | 82% | 84% | 82% | 77% | 80% |
| Female | 95 | 18% | 16% | 18% | 23% | 20% |
| **Age** |  |  |  |  |  |  |
| 18-24 | 147 | 29% | 24% | 29% | 40% | 25% |
| 25-34 | 204 | 40% | 40% | 38% | 40% | 47% |
| 35-44 | 107 | 21% | 27% | 20% | 10% | 22% |
| 45+ | 56 | 11% | 10% | 14% | 11% | 6% |
| **Marital status** |  |  |  |  |  |  |
| Married | 260 | 51% | 49% | 52% | 64% | 29% |
| Widowed or divorced | 31 | 6% | 4% | 5% | 11% | 10% |
| Single | 222 | 43% | 47% | 42% | 25% | 61% |
| No answer/refusal | 1 | 0% | 0% | 1% | 0% | 0% |
| **Asylum/refugee status** |  |  |  |  |  |  |
| Asylum process in Austria | 392 | 76% | 80% | 71% | 72% | 88% |
| Asylum seeking | 82 | 16% | 16% | 17% | 17% | 10% |
| Refugee (holding a convention passport), subsidiary protection or temporary suspension of deportation | 15 | 3% | 0% | 8% | 1% | 0% |
| No status yet | 23 | 4% | 4% | 3% | 10% | 2% |
| No answer/refusal | 2 | 0% | 0% | 1% | 0% | 0% |
| **Arrival in Austria** |  |  |  |  |  |  |
| January to August 2015 | 91 | 18% | 19% | 14% | 22% | 18% |
| September 2015 | 157 | 31% | 38% | 29% | 24% | 20% |
| October 2015 | 198 | 39% | 36% | 42% | 39% | 35% |
| November 2015 | 68 | 13% | 7% | 15% | 16% | 27% |
| **Religious affiliation** |  |  |  |  |  |  |
| Islam | 453 | 88% | 97% | 96% | 87% | 29% |
| Christian | 41 | 8% | 2% | 3% | 2% | 60% |
| None (e.g. Atheist) | 11 | 2% | 0% | 1% | 7% | 6% |
| Other | 5 | 1% | 1% | 0% | 2% | 2% |
| No answer/refusal | 4 | 1% | 1% | 0% | 1% | 4% |
| **Region of origin** |  |  |  |  |  |  |
| Baghdad | 99 | 19% | 51% |  |  |  |
| Basra | 20 | 4% | 10% |  |  |  |
| Nineveh | 18 | 4% | 9% |  |  |  |
| Other governorates in Iraq | 59 | 11% | 30% |  |  |  |
| Damascus ad suburbs | 55 | 11% |  | 30% |  |  |
| Aleppo | 38 | 7% |  | 21% |  |  |
| Homs | 22 | 4% |  | 12% |  |  |
| Other governorates in Syria | 67 | 13% |  | 36% |  |  |
| Kabul | 10 | 2% |  |  | 12% |  |
| Ghazni | 9 | 2% |  |  | 11% |  |
| Other provinces in Afghanistan | 20 | 4% |  |  | 24% |  |
| Regions not captured in detail | 51 | 10% |  |  |  | 100% |
| No answer/refusal | 46 | 9% | 0% | 1% | 53% |  |
| **Type of residence before coming to Austria** |  |  |  |  |  |  |
| Your own home | 162 | 31% | 28% | 38% | 24% | 35% |
| Your family’s home | 245 | 48% | 48% | 49% | 43% | 48% |
| A rental home | 106 | 21% | 24% | 13% | 33% | 17% |
| No answer/refusal | 1 | 0% | 1% | 0% | 0% | 0% |
| **Way to Austria** |  |  |  |  |  |  |
| Through Turkey | 499 | 97% | 100% | 99% | 93% | 85% |
| Through Libya | 1 | 0% | 0% | 0% | 0% | 2% |
| Other | 9 | 2% | 0% | 0% | 5% | 10% |
| No answer/refusal | 5 | 1% | 0% | 1% | 2% | 4% |
| **Average costs of travel per person** |  |  |  |  |  |  |
| Less than US$2,000 | 117 | 23% | 13% | 42% | 12% | 10% |
| US$2,000 to US$2,999 | 137 | 27% | 31% | 29% | 14% | 21% |
| US$3,000 to US$3,999 | 92 | 18% | 17% | 16% | 22% | 19% |
| More than US$4,000 | 111 | 22% | 26% | 8% | 34% | 33% |
| Much more | 39 | 8% | 9% | 4% | 12% | 8% |
| No answer/refusal | 18 | 3% | 4% | 1% | 6% | 10% |
| **Plans to return to the home country after the situation has stabilised** |  |  |  |  |  |  |
| Yes | 112 | 22% | 15% | 32% | 19% | 22% |
| No | 343 | 67% | 77% | 52% | 75% | 67% |
| Don’t know | 58 | 11% | 9% | 16% | 6% | 11% |
| No answer/refusal | 1 | 0% | 0% | 1% | 0% | 0% |
| Total | 514 | 100% | 100% | 100% | 100% | 100% |
| Total (n) | 514 |  | 196 | 184 | 83 | 51 |

Source: Displaced Persons in Austria Survey (DiPAS), n=514 interviewed persons.
