# Supplementary material for: Human Capital, Values, and Attitudes of Persons Seeking Refuge in Austria in 2015
Source: PLoS One. 2016 Sep 23;11(9):e0163481. doi: 10.1371/journal.pone.0163481 (PMC5035031; doi:10.1371/journal.pone.0163481)
Supplement: S2 Table — Source: Displaced Persons in Austria Survey (DiPAS), n = 1,391 individuals captured in the survey. (DOCX) [file pone.0163481.s006.docx]

#### S2 Table. Individuals captured in the sample.

|  | Respondents | Family in Austria | | | Family abroad | | |  |
| --- | --- | --- | --- | --- | --- | --- | --- | --- |
|  |  | Spouses | Children | | Spouses | Children | |  |
|  |  |  | Children 0-17 | Children 18+ |  | Children 0-17 | Children 18+ |  |
| Male | 419 | 50 | 156 | 34 | 19 | 135 | 20 | 833 |
| Female | 95 | 70 | 133 | 15 | 121 | 98 | 26 | 558 |
| Sum | 514 | 120 | 289 | 49 | 140 | 233 | 46 | 1,391 |
| Persons in Austria | 514 | 120 | 289 | 49 |  |  |  | 972 |
| Persons abroad |  |  |  |  | 140 | 233 | 46 | 419 |
| Sum | 514 | 458 | | | 419 | | | 1,391 |
| Estimation of potential for family reunification (i.e. persons abroad related to 972 the individuals in Austria) ^a^ |  |  |  |  | 0.14 | 0.24 | 0.05 | 0.43 |

Source: Displaced Persons in Austria Survey (DiPAS), n=1,391 individuals captured in the survey.

^a^ e.g.: 140/972=0.14
